# Activator Control of Nucleosome Occupancy in Activation and Repression of Transcription

**DOI:** 10.1371/journal.pbio.0060317

**Published:** 2008-12-23

**Authors:** Gene O Bryant, Vidya Prabhu, Monique Floer, Xin Wang, Dan Spagna, David Schreiber, Mark Ptashne

**Affiliations:** Molecular Biology Program, Sloan Kettering Institute, New York, New York, United States of America; University of Massachusetts Medical School, United States of America

## Abstract

The relationship between chromatin structure and gene expression is a subject of intense study. The universal transcriptional activator Gal4 removes promoter nucleosomes as it triggers transcription, but how it does so has remained obscure. The reverse process, repression of transcription, has often been correlated with the presence of nucleosomes. But it is not known whether nucleosomes are required for that effect. A new quantitative assay describes, for any given location, the fraction of DNA molecules in the population that bears a nucleosome at any given instant. This allows us to follow the time courses of nucleosome removal and reformation, in wild-type and mutant cells, upon activation (by galactose) and repression (by glucose) of the *GAL* genes of yeast. We show that upon being freed of its inhibitor Gal80 by the action of galactose, Gal4 quickly recruits SWI/SNF to the genes, and that nucleosome “remodeler” rapidly removes promoter nucleosomes. In the absence of SWI/SNF, Gal4′s action also results in nucleosome removal and the activation of transcription, but both processes are significantly delayed. Addition of glucose to cells growing in galactose represses transcription. But if galactose remains present, Gal4 continues to work, recruiting SWI/SNF and maintaining the promoter nucleosome-free despite it being repressed. This requirement for galactose is obviated in a mutant in which Gal4 works constitutively. These results show how an activator's recruiting function can control chromatin structure both during gene activation and repression. Thus, both under activating and repressing conditions, the activator can recruit an enzymatic machine that removes promoter nucleosomes. Our results show that whereas promoter nucleosome removal invariably accompanies activation, reformation of nucleosomes is not required for repression. The finding that there are two routes to nucleosome removal and activation of transcription—one that requires the action of SWI/SNF recruited by the activator, and a slower one that does not—clarifies our understanding of the early events of gene activation, and in particular corrects earlier reports that SWI/SNF plays no role in *GAL* gene induction. Our finding that chromatin structure is irrelevant for repression as studied here—that is, repression sets in as efficiently whether or not promoter nucleosomes are allowed to reform—contradicts the widely held, but little tested, idea that nucleosomes are required for repression. These findings were made possible by our nucleosome occupancy assay. The assay, we believe, will prove useful in studying other outstanding issues in the field.

## Author Summary

In this paper, we examine activation and repression of transcription of a gene in yeast. This gene, like the typical human gene, is wrapped in DNA-protein packets called nucleosomes. It is widely believed that these condensed packets are unwrapped, in a process called nucleosome removal, as transcription begins. Here, we describe a new quantitative nucleosome assay that allows us to measure the time course of nucleosome removal and replacement as the gene is activated and repressed. The yeast activator Gal4, bound to DNA, effects activation of gene transcription in two separate steps. First, it recruits to the gene an enzyme that strips off nucleosomes; and second (as we had shown previously), it recruits the transcriptional machinery. We also show that transcription of the gene can be turned off well before nucleosomes have been returned to the gene. In this case, the activator continues to recruit the nucleosome-remover, but either the transcriptional machinery is not recruited, or if it is, it is soon destroyed

## Abstract 3

A new nucleosome-occupancy technique reveals how the transcriptional activator Gal4 determines chromatin structure as genes are activated and repressed.

## Introduction

Gal4 is an intensively studied transcriptional activator found in the yeast Saccharomyces cerevisiae. Galactose, added to the growth medium, frees Gal4 of its inhibitor Gal80, and the DNA-bound activator quickly and strongly induces genes required to metabolize the sugar. Two such genes are the divergently transcribed *GAL1* and *GAL10*, between which lie four Gal4 binding sites comprising the so-called upstream activating sequence, galactose (*UASg*). A wide array of studies shows that Gal4 recruits to nearby yeast genes protein complexes required for transcription [1,2]. Gal4 also activates any of a wide array of genes in higher eukaryotes when ectopically expressed, provided the target gene bears Gal4 binding sites nearby. This ability to activate so many genes in so many different organisms probably reflects its ability to bind, and thereby recruit, a wide array of targets. For example, Gal4 contacts at least three yeast protein complexes (called SAGA, TFIID, and Mediator) [3,4], and thereby activates transcription of genes that require different subsets of these complexes [5,6]. Addition of glucose, a preferred carbon source, to cells growing in galactose inhibits expression of the *GAL* genes in several ways. The strongest direct effect is repression of *GAL4* and of *GAL2*, which encodes the galactose permease. A smaller effect is that the *GAL1,10* genes are also directly repressed (see Discussion) [7–10].

Gal4, like other eukaryotic transcriptional activators, must work despite the fact that DNA is wrapped in nucleosomes. For example, nucleosomes in the *GAL1* and *GAL10* promoters (referred to as promoter nucleosomes) would cover DNA that must be available for the transcriptional complex to form. And indeed, several experiments show that these nucleosomes, present on the inactive promoters, are missing when the genes are transcribed [11–17]. One mechanism for this nucleosome loss would be that the recruited machinery simply competes them away. Consistent with this idea, fusion proteins bearing a DNA binding domain attached to one or another subunit of the transcriptional machinery (e.g., LexA-Gal11) can activate transcription to a high level. Such fusion proteins presumably directly recruit the transcriptional machinery to the promoter without removing nucleosomes in a separate step [18]. A second possibility for nucleosome removal by an activator would be that it recruits a function that removes nucleosomes in a step separate from recruitment of the transcriptional machinery itself. This scenario has been shown to hold for the *PHO8* gene of yeast: in this case, the nucleosome remodeling complex SWI/SNF, recruited to a gene by the activator, removes promoter nucleosomes in an early step in the process of gene induction [19]. It has been reported, however, that SWI/SNF plays no role in ordinary induction of the *GAL* genes [20,21].

Just as removal of promoter nucleosomes is correlated with activation of transcription, so is their reformation typically correlated with the turning off of transcription. For example, when cells are transferred from galactose to glucose, and *GAL* gene transcription ceases, promoter nucleosomes rapidly reform at these genes [15,17]. Whether this reformation of promoter nucleosomes is required for gene silencing, or rather is a consequence of that inactivity, is not known. In the typical analysis of glucose repression of the *GAL* genes, Gal4 is either inactive (due to the absence of galactose) or is depleted by one of the long-term effects of glucose as mentioned above. The possibility that Gal4 might continue to function in the simultaneous presence of galactose and glucose, and if so to what end, has not, to our knowledge, heretofore been considered.

Here, we reexamine these matters using a quantitative micrococcal nuclease protection assay that measures, at any given moment, and for any specified DNA fragment, the fraction of the population that is occupied by a nucleosome in vivo. We show that in an early step of gene activation, Gal4 recruits SWI/SNF and quickly removes promoter nucleosomes. In the absence of SWI/SNF, a high level of transcription is also reached and promoters cleared of nucleosomes, but the process proceeds significantly more slowly. We confirm that upon transferring cells from galactose to glucose, transcription quickly diminishes, and nucleosomes rapidly reform at the promoter [17]. In contrast, however, if glucose is added to cells growing in galactose, although mRNA production also quickly diminishes, nucleosomes do not rapidly reform. We show that, under these repressive conditions, galactose continues to counter the inhibitory effect of Gal80. Gal4 continues to recruit SWI/SNF, which, in turn, prevents nucleosome reformation despite the onset of repression.

## Results

### Nuclease Sensitivity in and around the *GAL1,10 UASg*

It has long been recognized that nucleosomes protect from micrococcal nuclease digestion fragments of DNA of about 140–160 bp. In a typical modern version of such an experiment, cells are fixed with formaldehyde, and isolated chromatin is lightly digested with a single dose of nuclease for a fixed time. Cross-linking is then reversed, and mononucleosomal-sized DNA fragments of about 150 bp are isolated. These recovered fragments are identified by PCR, microarray analysis, and/or DNA sequencing [22–24]. The novel aspect of our assay is that cross-linked chromatin is digested with nuclease over a wide range of nuclease concentrations. We then use a series of overlapping primer pairs (amplicons, ∼60 bp each) to quantitate the reaction products by real-time PCR without any prior fractionation for the size of protected fragments. Our method, like others, allows us to delineate arrayed nucleosomes (as confirmed by chromatin immunoprecipitation [ChIP] analysis) and to distinguish them from randomly positioned nucleosomes. Beyond that, however, we shall see that for any DNA segment potentially bearing a positioned nucleosome (such as in the *GAL1* promoter), we can determine the fraction of that DNA segment, in the population, that is occupied by a nucleosome at the moment of cross-linking. This allows us to determine nucleosome positioning and occupancy prior to induction and to follow the time courses of nucleosome removal and reformation upon induction and repression. Consistent with many previous studies using micrococcal nuclease (e.g., [11–13]), the transcriptional machinery itself, recruited to the promoter, does not protect against micrococcal nuclease digestion. (see Figure S1).

**Figure 1:**
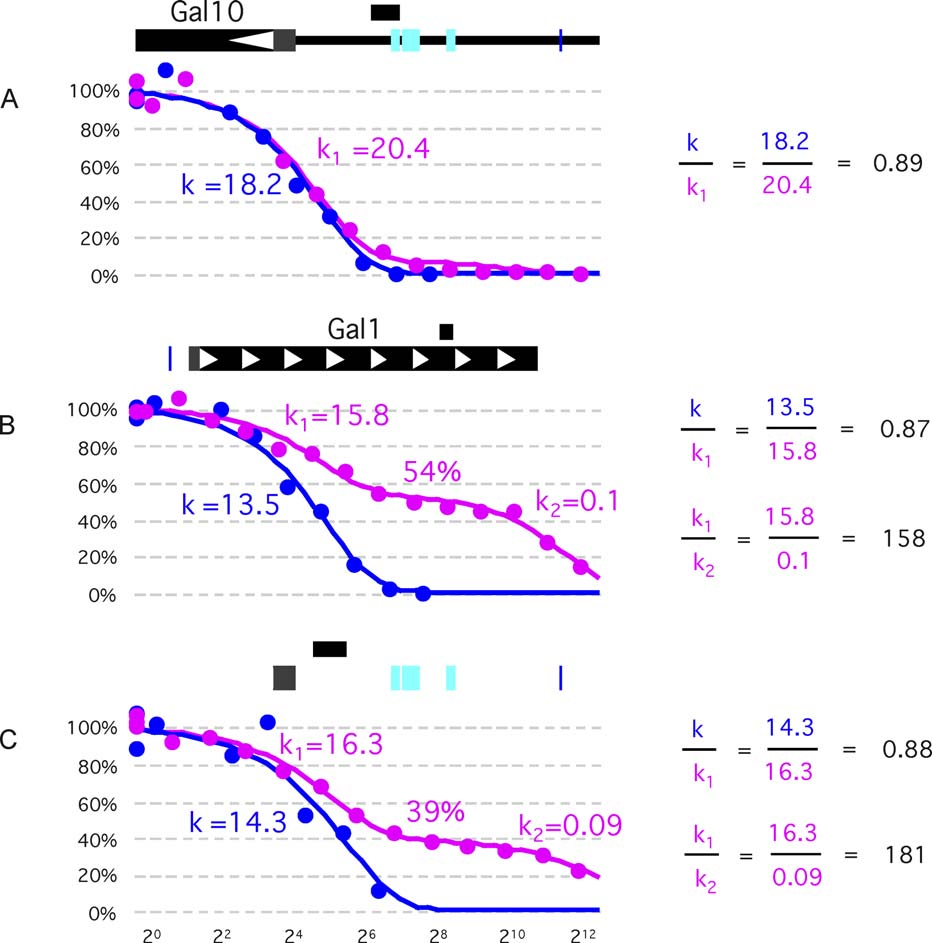
Micrococcal Nuclease Digestion of Purified DNA (Blue) and In Vivo Cross-Linked Chromatin (Magenta) The short black bar above each schematic identifies the approximately 60-bp region analyzed in each case. Plotted is the percentage DNA (*y*-axis), analyzed by real-time PCR, remaining after a fixed time of exposure to a specified concentration of micrococcal nuclease as indicated in arbitrary units along the *x-*axis. The curves show the best fit to a one-state or two-state exponential decay function. For each of the three cases, *k* is the rate of digestion of segments analyzed in purified DNA (blue curves). For the segments analyzed in cross-linked chromatin, *k*_1_ describes the faster-digested portion and *k*_2_ the slower-digested portion of the biphasic curves. In (A), there is essentially no protected fraction, and therefore no *k*_2_. The *k* values for purified DNA were normalized to adjust for different conditions in different experiments (e.g., specific activity of the nuclease) (see main text). (A) Shows digestion of a chromatin fragment bearing a “hypersensitive site” (magenta) and its counterpart in purified DNA (blue). The DNA segment lies immediately adjacent to the *UASg* as indicated by the black bar above the schematic. Gal4 binding sites are indicated in cyan. (B and C) show typical biphasic curves describing digestion of DNA segments in which a fraction of the population of each segment registers naked and another fraction occupied. The fraction occupied (by a nucleosome) is indicated as 54% and 39% in the two cases. The DNA segment analyzed in (B) is found in the *GAL1* ORF; and that of (C) is taken from an uninduced *GAL10* promoter.

In initial experiments, we found, as expected, that DNA, purified from cells not exposed to the cross-linking reagent (hereafter referred to simply as purified DNA), yielded, in every case, a first-order decay function when digested and analyzed as outlined above. The digestion rates of the segments (*k*, blue lines in Figure 1) varied in value as much as 10-fold, indicating differences in the intrinsic sensitivities of different DNA sequences to micrococcal nuclease. When this experiment was performed on cross-linked chromatin, a few locations yielded a monophasic digestion pattern like that of purified DNA, indicating the presence of a single species in the population. The absolute values of the relevant digestion rates varied from experiment to experiment depending upon the specific activity of the nuclease, DNA concentrations, and impurities in the chromatin preparations. We normalize these digestion rates by setting that of one of these locations (which we call naked, or hypersensitive [HS]) equal to that of its counterpart in purified DNA. We then find that the rates of digestion of the other HS sites, compared to the first, are predicted by their relative intrinsic sensitivities as determined with purified DNA (see Figure 1A for an example). Exceptions to this rule—that the digestion rate describing a monophasic curve is predicted by the digestion rate of the corresponding purified DNA—are found in the *UASg*, a matter we return to below.

Most chromatin locations yield curves that, unlike those just discussed, are biphasic, consisting of rapidly digested and slowly digested portions, indicating the presence of two subpopulations. Considering only the rapidly digested portion, again the first order rates of the reaction were related to one another as were those of their counterparts in purified DNA. We call this subpopulation naked. In striking contrast, for the remaining portion of each biphasic curve, the digestion rate (again first order) was some 200-fold slower than that in the faster digesting portion (compare *k*_1_ and *k*_2_ in Figure 1B and 1C). The fact that this degree of protection is so constant over so many locations (varying no more than plus or minus some 2-fold) suggests that it is caused by a common factor bound to DNA. As this and other evidence confirms, the typical protecting factor is a nucleosome. Thus, analysis of each biphasic curve reveals, for the corresponding DNA fragment, the fraction in the population that bears a nucleosome, and the fraction naked, at the moment of cross-linking. For example, the chromatin fragment of Figure 1B comprises a population about 46% naked and 54% occupied. For the case of Figure 1C, the corresponding fractions are 61% and 39%. Of the approximately 500 DNA segments we have examined from around the genome, most yield biphasic digestion curves, and these curves differ from each other primarily in the percent protected as in the examples shown.

### The *GAL* Genes

**Figure 2:**
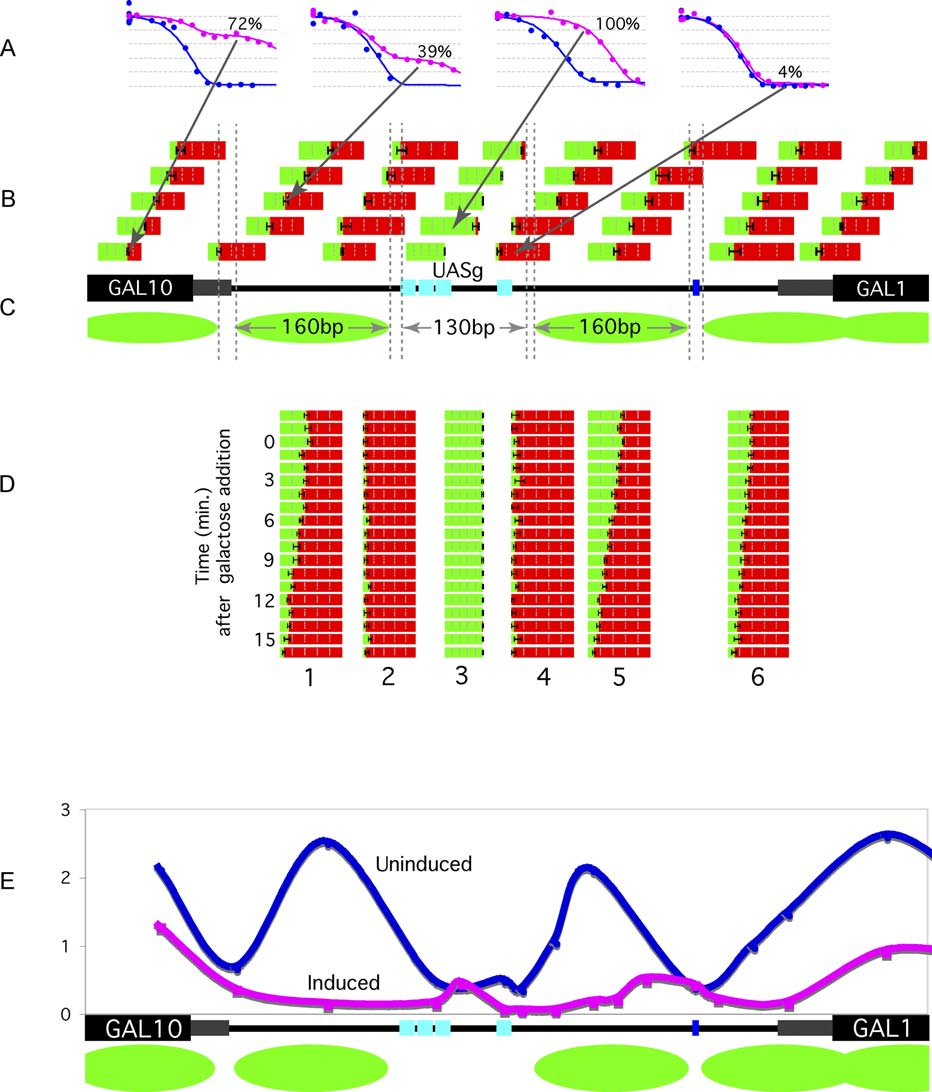
Nuclease Protection Pattern at the *GAL1,10* Locus Prior to (A and B) and Following Addition of Galactose (D) and ChIP Analysis (E) (A) The curves describe digestion of four DNA fragments as in Figure 1 for cells growing in raffinose. The arrows indicate, for each case, the fragment analyzed. As in Figure 1, in each case, the blue curve describes purified DNA, and the magenta curve in vivo cross-linked DNA. The percent protected (occupied) is indicated for each case. (B) The position of each bar identifies the in vivo cross-linked DNA segment analyzed as described in the main text for cells growing in raffinose. The fraction of each bar that is green represents the fraction of the segment, among the population, that is occupied. Double vertical hatched lines indicate hypersensitive sites as defined in the main text. The distances between hypersensitive sites in base pairs are indicated. (C) In this schematic of the *GAL1,10* locus, the black rectangles represent the two ORFs, and the gray rectangles the regions between the start sites of transcription and translation. The cyan rectangles are Gal4 binding sites, and the blue rectangle is a TATA box. Green ovals represent nucleosomes. (D) Addition of galactose to cells growing in raffinose triggers the changes in nuclease protection described in this figure. On the left are listed minutes following addition of galactose. Six representative segments are displayed. As nucleosomes are removed, the fraction of the corresponding bars that is green decreases (indicating an ever-decreasing fraction of the population of this fragment that is occupied), whereas the bar from the *UASg* remains green throughout. (E) A ”high-resolution” ChIP experiment was used to probe for FLAG-tagged histone H2B in uninduced and induced cells. Cells were grown in glucose (blue line) and galactose (magenta line). The *x*-axis denotes the *GAL1,10* locus and the *y*-axis denotes the immunoprecipitated H2B signal normalized to a region at telomere VI. The positions of the histone as assayed by ChIP are presented above the same schematic as in (C).

Figure 2 shows that in cells in which the *GAL1,10* genes were not expressed, rather precisely positioned nucleosomes were found flanking the *UASg*. This conclusion was reached, in part, by experiments in which we measured the nuclease sensitivities of many overlapping short (∼60 bp) DNA segments. The nuclease sensitivities of 40 such segments are represented by the colored bars above the gene schematic (Figure 2B). Each bar represents a single DNA fragment, indicated by its position, assayed using a specific amplicon. The fraction of each bar that is green corresponds to the fraction of the population of the corresponding DNA fragment that was protected (occupied) in a typical experiment, and vice versa for the fraction that is red. Thus, each bar represents a digestion curve, four examples of which are shown in Figure 2A. Whenever a bar crosses one of the paired vertical dashed lines, it is largely red, indicating that that site is naked (unprotected, HS) in essentially every member (>90%) of the population in vivo. About 130 bp (plus or minus a few base pairs) separate the two HS sites flanking the *UASg*, but 160 bp separate the HS sites to the right and left of the *UASg*. Two less well-defined HS sites lie further downstream and upstream, separated again from their neighboring HS sites by about 160 bp.

The repeat length of 160 bp is that expected if nucleosomes are positioned as shown in Figure 2C (green ovals), each protecting about 160 bp from digestion and separated from each other by 10–15 bp. The ChIP experiment of Figure 2E, in which FLAG-tagged H2B was probed, supports the conclusion that the 160-bp regions between HS sites in Figure 2 are occupied by nucleosomes. This experiment (a “high-resolution” ChIP) included a step in which cross-linked chromatin was lightly digested with micrococcal nuclease prior to immunoprecipitation [25]. Absent this step, the nucleosome positioning is less well defined (see Figure S2). These nucleosomes are positioned on DNA sequences crucial for formation of the transcription complex. Thus the two nucleosomes on the *GAL1* promoter around the TATA box, and the single *GAL10* promoter nucleosome span the distance between the *UASg* and the transcription start site. In the remainder of this paper, we refer to the nucleosome positioned just to the right of the *UASg* in Figure 2C as the *GAL1* upstream nucleosome, and to the three nucleosomes of the figure as promoter nucleosomes. The results of these micrococcal nuclease digestion experiments and the ChIP experiments were essentially the same whether cells were grown in raffinose (a noninducing sugar) or glucose. The depicted positions of these nucleosomes are consistent with earlier analyses [11,14,16].

Figure 2B also shows that as indicated by the positions of the largely or completely green bars, prior to induction, DNA segments in the *UASg* were protected in essentially every member of the population. Several experiments indicate that this protection is caused by some molecule bound to the *UASg* and that this molecule is neither Gal4 nor a nucleosome. First, the *UASg* isolated from cross-linked chromatin (see Figure 2A) is digested more slowly than predicted from the rate of digestion of its purified counterpart, indicating that the nuclease resistance of this fragment is not an intrinsic property of its sequence. Second, the pattern of protection shown in Figure 2B, obtained with wild-type cells grown in raffinose, was unchanged by deletion of *GAL4* (unpublished data). Third, as noted above, the ChIP experiment of Figure 2E shows the region to be free of histone H2B. Fourth, the rate of digestion of the protected fragment was significantly faster than that predicted were the protecting factor a nucleosome (see Figure S3). Fifth, as shown in Figure 2C, the size of the protected *UASg* fragment, defined by the distance separating the flanking HS sites, is considerably smaller than that of the repeat length of a nucleosome (130 bp versus 160 bp). Finally, as we shall see (Figure 2D), the promoter nucleosomes are removed upon induction, rendering the DNA naked in our assay, whereas the *UASg* remains protected throughout our experiments. We do not know the identity of the putative molecule bound to the UASg, nor do we know its function, if any. Others have noted that some molecule other than a nucleosome or Gal4 can occupy the *UASg* [14,26,27]. We draw attention to this molecule here only because, as we shall see, the protection it confers, which remains constant throughout our experiments, serves as a useful reference point.

We return now to the nucleosomes flanking the *UASg*. Figure 2D shows that at the moment of cross-linking, a significant fraction of the population is missing one or another of the depicted nucleosomes prior to induction. Thus, columns 1, 5, and 6, which represent DNA segments found at the centers of the three positioned nucleosomes, show that prior to induction (the top three rows), only about half of each segment in the population is protected. In contrast, as shown in column 3, the *UASg* is 100% protected. Although more complicated scenarios might be imagined (see Discussion), a simple explanation for these results is that at any given instant, one or more of the depicted nucleosomes is absent from about 50% of the population. This level of occupancy was essentially the same in wild-type cells grown in either glucose or raffinose, and was unchanged by deletion of *GAL4* (unpublished data) . Our experiments, as well as those of others, indicate a nucleosome disposition in the *GAL1* ORF different from that found in the promoter. Thus, ChIP experiments indicate the presence of histones more or less uniformly across the ORF (see [28] and Figure S4) Because the nucleosomes in the ORF are not regularly positioned as they are in the promoter, it is difficult to measure precisely the typical level of occupancy of an individual ORF nucleosome. We do, however, estimate that level to be significantly higher in the ORF than in the promoter (see Figure S4).

### Induction by Galactose

**Figure 3:**
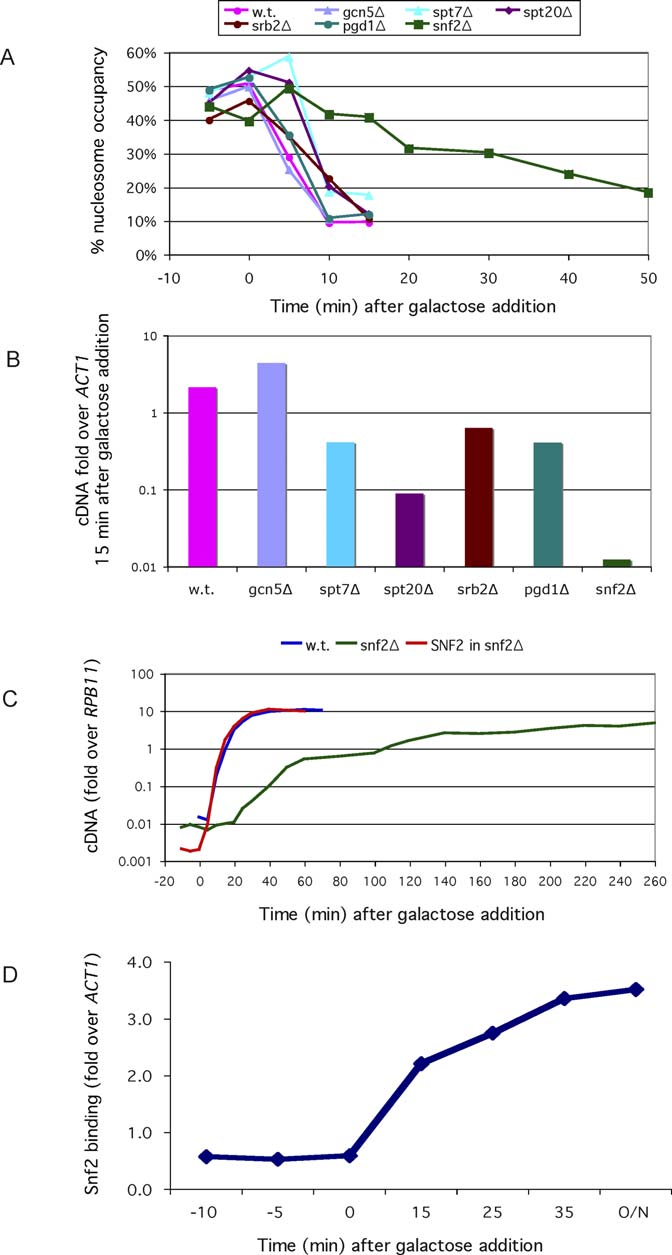
Recruitment and Function of SWI/SNF upon Induction (A) The presence of the *GAL1* upstream nucleosome is plotted as a function of the time after addition of galactose to cells growing in raffinose. The experiment was performed as in Figure 2D for a wild-type strain and for each of several deletion mutants as indicated. (B) The level of *GAL1* mRNA at 15 min following addition of galactose is indicated for the wild-type and mutant strains. (C) The level of *GAL1* mRNA at various times following induction is shown for three strains: a wild-type strain (BY4741–blue line); its *snf2* deletion derivative (green line); and the *snf2Δ* mutant to which a wild-type *SNF2* allele was added on a plasmid (red line). (D) The level of myc-tagged Snf2 at the *GAL1* promoter, assayed by ChIP, is shown as a function of time after addition of galactose. O/N indicates an overnight culture.

Figure 2E shows that as analyzed by a ChIP experiment, *GAL1* promoter nucleosomes, present before induction, were absent from cells grown for many generations in galactose. The time course of removal of these nucleosomes following the addition of galactose to cells growing in raffinose is revealed by our protection assay (Figure 2D). Consider, for example, the fragments represented in columns 1, 5, and 6 in the figure. For each of these fragments, the fraction of the population occupied by a nucleosome steadily decreased as induction proceeded. Nucleosome removal began about 5 min after addition of galactose and was complete by about 12–16 min. In contrast, the *UASg* remained highly protected. The naked regions flanking the *UASg*, as well as that separating the two nucleosomes to the right of the *UASg*, remained naked. An induction experiment in which we simultaneously measured nucleosome removal (using the nuclease protection assay) and recruitment of the transcriptional machinery to the *GAL1* promoter (using the ChIP assay as in [1]), showed that nucleosome removal was approximately coincident with the appearance at the promoter of the transcriptional machinery. We also found that as previously reported [29], SWI/SNF is quickly recruited to the *UASg* by Gal4 (Figure 3D).

Figure 3A shows the progressive removal of the *GAL1* upstream nucleosome in a series of mutant strains. The figure shows that deletion of the SAGA component *SPT20*, which drastically reduces formation of the transcription complex [1,30,31], had no effect on the time course of nucleosome removal. Deletion of any of the following genes also had no effect on the rate of this reaction: *GCN5*, which encodes the histone acetyltransferase in SAGA; *SPT7*, which encodes a core SAGA component; and *SRB2* and *PGD1*, which encode Mediator components. The figure also shows, in striking contrast, that deletion of *SNF2*, the catalytic subunit of the SWI/SNF complex, dramatically delayed nucleosome removal and the onset of transcription (Figure 3C). Figure 3B shows that certain mutations that had no effect on nucleosome removal nevertheless had strong deleterious effects on transcription. Thus, at 15 min postinduction, *GAL1* mRNA levels were strongly diminished in the *spt20*Δ and *snf2*Δ strains, moderately diminished in the *spt7*Δ, *pgd1*Δ, and *srb2*Δ strains, and essentially at wild-type levels in the *gcn5*Δ strain. The other two promoter nucleosomes behaved identically to the *GAL1* upstream nucleosome in these experiments (G. O. Bryant and M. Ptashne, unpublished data). Figure 3C, a time course of mRNA production following induction, shows that in *snf2*Δ mutants, mRNA production reached levels obtained with the wild-type strain, but only over a much longer time course that paralleled promoter nucleosome loss. These experiments also support the finding alluded to above that the recruited transcriptional machinery, readily detected by ChIP assays, does not protect against micrococcal nuclease digestion.

The conclusion that SWI/SNF is required for rapid nucleosome removal, and that delayed nucleosome removal also delays the onset of transcription, stands in contrast to a report that mutation of *SNF2* has no effect on induction of transcription of the *GAL* genes [20]. We therefore obtained the *snf2*Δ strain of Kundu et al., and found that this strain, as well as the *snf2*Δ strain to which a wild-type SNF2 allele had been added, behaved identically to our corresponding strains in an assay for the rate of synthesis of mRNA upon induction (Figure S5).

### Glucose Repression

**Figure 4:**
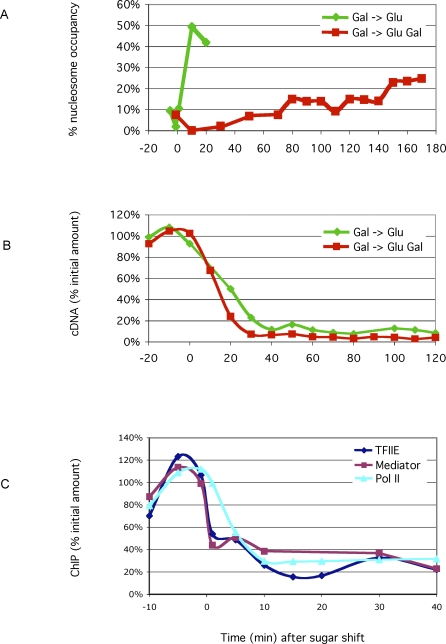
Nucleosome Reformation, mRNA Production, and Binding of the Transcriptional Machinery as Affected by Glucose (A) The presence of the *GAL1* upstream nucleosome was assayed in the nuclease protection assay, as described in the main text, following two changes in the growth medium. The times in minutes after the medium changes are indicated below the graph. Green line: cells growing in galactose were spun down, washed, and then transferred to medium lacking galactose, but containing 2% glucose. Red line: the washed cells were in this case transferred to medium containing 2% galactose plus 2% glucose. (B) The levels of *GAL1* mRNA were measured at various times after the media changes described in (A). The green and red lines correspond to growth conditions as in (A). (C) The levels of three components of the transcriptional machinery at the *GAL1* promoter were measured at various times as indicated following transfer of cells growing in galactose to medium containing galactose and glucose. The three proteins measured by ChIP analysis as in [1] were Tfa2 (blue), Gal11 (dark magenta), and Rpb1 (cyan). Tfa2 is a component of TFIIE, Gal11 of Mediator, and Rpb1 of RNA polymerase II.

Figure 4A shows that in cells grown in galactose, washed, and then resuspended in glucose, the *GAL1* upstream nucleosome quickly (within 10 min) reformed on a fraction of templates equal to that occupied prior to induction. The other two nucleosomes in Figure 2 reformed with an indistinguishable time course (G. O. Bryant and M. Ptashne, unpublished data). This time course of promoter nucleosome reassembly mirrored the time course of loss of mRNA production (Figure 4B, green line).

A strikingly different result was obtained if, instead of transferring cells from galactose to glucose, the cells were resuspended in medium containing glucose (2%) plus galactose (2%). Figure 4A (red line) shows that in this case, over the first few hours following this transfer, nucleosomes reformed only slowly. As indicated in Figure 4B, however, transcription decreased as dramatically as in the previous experiment. Thus, for example, the early phase of glucose repression (as assayed by mRNA production in Figure 4B) was complete by 30 min, but nucleosome formation only reached about half of its original value by 3 h. Figure 4C, a ChIP experiment, shows that several components of the transcriptional machinery (RNA polymerase II, Gal11, and TFIIE), each of which had bound to the promoter in cells grown in galactose, were quickly depleted from the promoter upon resuspension in glucose plus galactose, and the time course of this depletion mirrored the time course of repression of transcription. ChIP analysis also showed that Gal4 was bound to the *UASg* over the course of the experiments of Figure 4 (Figure S6). The glucose repression of the *GAL* genes we observed upon transfer to glucose plus galactose was not due to a nonspecific effect on transcription. Thus, three other genes (*HHF1*, *ACT1*, and *RPB11*), which are constitutively active, remained so in the presence of glucose in our experiments (unpublished data).

**Figure 5:**
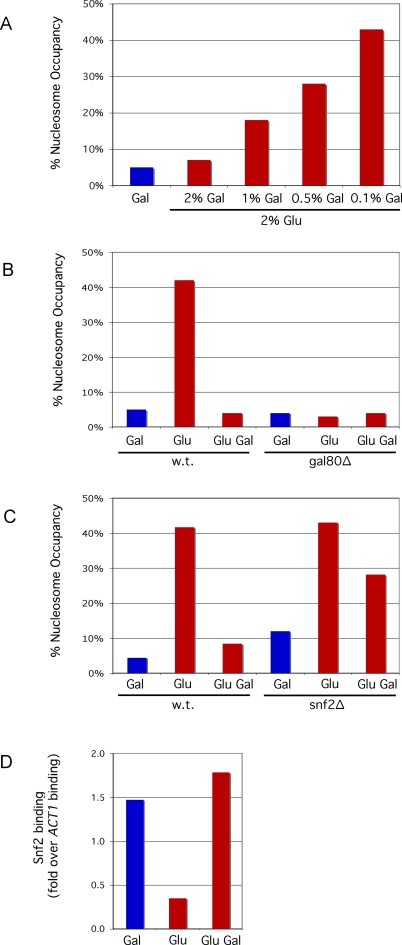
The Effect of Galactose and Recruited SWI/SNF on Nucleosome Reformation in the Presence of Glucose (A) The presence of the *GAL1* upstream nucleosome was measured in cells grown overnight in 2% galactose (blue bar) and 20 min following resuspension of these cells in 2% glucose plus the indicated levels of galactose (red bars). (B) The presence of the *GAL1* upstream nucleosome was assayed in wild-type (w.t.) and *gal80Δ* strains. For both strains, the cells were grown overnight in 2% galactose (blue bars) and then transferred for 20 min to medium containing either 2% glucose or 2% glucose plus 2% galactose (red bars) as indicated. (C) The presence of the *GAL1* upstream nucleosome was assayed in wild-type and *snf2Δ* strains. For both strains, the cells were grown overnight in 2% galactose (blue bars) and then transferred for 60 min to medium containing either 2% glucose or 2% glucose plus 2% galactose (red bars) as indicated. (D) The level of Snf2 at the *GAL1* promoter was assayed as in Figure 3D in cells grown overnight in 2% galactose (blue bar) and then 60 min following transfer to medium containing either 2% glucose or 2% glucose plus 2% galactose as indicated (red bars).

Figure 5A shows that although repression of transcription by glucose was not affected by the simultaneous presence of galactose, nucleosome reassembly was. Thus, at some 20 min after addition of glucose, the extent of nucleosome reassembly was approximately inversely proportional to the concentration over a 20-fold range, of galactose. The results indicate that even in the presence of glucose, galactose activates Gal4 (by removing the inhibitory effect of Gal80), and Gal4 recruits some function that keeps the region nucleosome-free despite the repression of transcription. The following experiments support this surmise. First, ChIP experiments show that Gal4 remained present at the *UASg* over these time courses (Figure S6). Second, as shown in Figure 5B, in the absence of Gal80, galactose had no effect on the extent of nucleosome redeposition. In this case, Gal4 worked constitutively to keep the promoters nucleosome-free even though transcription was repressed.

And finally, two additional experiments involving glucose repression identify a crucial function recruited by Gal4 as—once again—SWI/SNF. First, the ChIP experiment of Figure 5D shows that following resuspension in medium containing galactose and glucose, Snf2 continued to be recruited to the promoter, whereas if glucose was substituted for galactose, Snf2 recruitment ceased. Second, we induced *snf2*Δ mutant cells by growth in galactose (a process that takes many hours to be fully realized—see Figure 3C), and then resuspended the cells in medium containing glucose and galactose. In this case, nucleosomes reformed more quickly than in wild-type cells (see Figure 5C). We conclude that recruited SWI/SNF plays a significant role in keeping the promoter nucleosome-free following transfer of cells from galactose to medium containing galactose and glucose. These experiments were also performed simply by adding glucose to cells growing in galactose, with results essentially the same as those obtained by resuspending cells in glucose plus galactose (unpublished data).

## Discussion

Our experiments, taken with other work, describe the following scenarios. Prior to induction, nucleosomes positioned so as to compete for binding with the recruited transcriptional machinery can be found on *GAL1,10* promoters. At any given instant prior to induction, one or more of these nucleosomes are absent from a significant fraction of the promoters. In the absence of SWI/SNF, Gal4 recruits the transcriptional machinery, which evidently competes away the remaining nucleosomes and elicits a high level of transcription. Nucleosome removal and the onset of transcription occur significantly more quickly, however, if SWI/SNF is available to be recruited by Gal4. Thus, both of the scenarios for Gal4-mediated removal of promoter nucleosomes sketched in the Introduction can be realized. Gal4 can also effect nucleosome disposition under repressive conditions. Thus, addition of glucose to cells growing in galactose immediately represses transcription, but Gal4 continues to work, recruiting SWI/SNF and keeping the promoter nucleosome-free. In contrast, when cells are transferred out of galactose (thereby inactivating Gal4) and into glucose, promoter nucleosomes rapidly reform. Most of our findings were made possible by our quantitative micrococcal nuclease protection assay that measures fractional nucleosome occupancy in the population for any given DNA site. We now further discuss some of these matters.

### Induction

Our finding that none of several components of the transcriptional machinery is required for rapid removal of promoter nucleosomes indicates that the action of SWI/SNF, recruited by Gal4 and perhaps assisted by chaperones [32], suffices to remove nucleosomes in an early step of transcriptional activation. In several strains mutated for components of SAGA and the Mediator, mRNA production was severely delayed or diminished with no effect on nucleosome removal. We do not know whether histone acetylation aids in this reaction, but we saw no difference in the rate of nucleosome removal between wild-type cells and cells deleted for the histone acetyltransferase *GCN5*. Our results also show that in the absence of SWI/SNF, nucleosome removal and transcription were elicited by the action of Gal4, but the reactions were considerably slower than in the presence of SWI/SNF (requiring hours versus minutes). In this case, assuming the recruited machinery competes away the promoter nucleosomes, the reaction may be facilitated by the fact that these nucleosomes spontaneously vacate the promoter relatively frequently (see Promoter Nucleosomes below). Such a scenario might also explain the ability of fusion proteins, bearing a DNA binding domain attached to a component of the transcriptional machinery (e.g., LexA-Gal11), to activate transcription to a high level. Consistent with this idea, we have found that activation by such a fusion protein follows a slow time course approximating that triggered by Gal4 in a strain mutant for SWI/SNF (X. Wu, M. Floer, and M. Ptashne, unpublished data). A previous claim, emanating from this laboratory, that SWI/SNF is not involved in induction of the *GAL* genes [21], is explained by a failure to examine the stages in the course of the reaction, relying instead upon the end result observed after many hours of induction. In a different report, analyzed in the text, it was claimed that SWI/SNF had no effect on the onset of transcription [20]. Our results are in contrast to that claim.

### Glucose Repression in the Absence of Promoter Nucleosomes

We had no reason to anticipate our finding that galactose can continue to signal to Gal80, removing its inhibitory effect on Gal4, even as transcription is repressed by glucose. This was first suggested by the observation that the extent of nucleosome reformation some 30 min following addition of glucose was approximately inversely proportional to the concentration in the medium of galactose. This suggestion was confirmed by our finding that in a strain deleted for *GAL80*, the promoter remained nucleosome-free at this time point even in the absence of galactose. We surmised that Gal4 maintains the promoter nucleosome-free under repressive conditions just as it does when activating transcription, namely, by recruiting SWI/SNF. As predicted by this scenario, in a strain mutated for SWI/SNF, promoter nucleosomes rapidly reformed upon addition of glucose whether or not galactose remained present.

The ability of Gal4 to work at early times following the addition of glucose, maintaining the promoters nucleosome-free, does not contradict the known mechanisms for glucose repression alluded to in the Introduction. Thus, direct repression of *GAL4* and *GAL2*, even if immediate, would have an effect on *GAL1,10* expression only as the previously synthesized Gal4 and Gal2 proteins were diluted away. And direct repression of *GAL1,10*, as measured hours after addition of glucose, is reported to be weak, only some 2–3-fold [7,9]. This direct repression is believed to be caused by recruitment of the Tup1 repressing complex to the *GAL1,10* region by the specific DNA binding protein Mig1, and in preliminary experiments, we have found little if any alleviation of early glucose repression of *GAL1,10* by deleting *MIG1* (G. O. Bryant and M. Ptashne, unpublished data). As expected from these various considerations, in our experiments, nucleosomes do slowly reform at promoters in the presence of galactose and glucose, presumably a consequence of depletion of Gal4 and Gal2.

An implication of our findings is that early negative effects of glucose on transcription cannot be ascribed to an elimination of all Gal4 recruiting activities. We do not know whether other recruiting activities of Gal4 remain functional, but if so, it is possible that glucose somehow causes destruction of any transcription complex that might be recruited to the promoter. This notion would be consistent with previous suggestions that certain mutations in that complex can diminish the negative effects of glucose [33–36].

### Promoter Nucleosomes

Our analysis has equated protection from nuclease digestion with nucleosome occupancy (excluding the exceptional case of the *UASg*). Thus, for example, where we find DNA locations that yield biphasic digestion curves, indicating two subpopulations, we have identified the slow digesting fraction as occupied by a nucleosome, the fast digesting portions as naked, i.e., simply lacking nucleosomes. And, we have argued, that the progressive increase in the fraction naked, as induction proceeds, reflects nucleosome loss. A more complicated description for the naked fraction could be imagined. Thus, for example, perhaps prior to induction, the naked regions bear nucleosomes in some aberrant configuration that would expose a segment of DNA so as to render it “naked” in our experiments, and according to this notion, as induction proceeds, instead of falling off, the nucleosomes increasingly adopt that aberrant configuration. It is difficult to completely exclude such a scenario. However, our ChIP analysis, which probed for histone H2B (Figure 2E), as well as ChIP analyses probing additionally for histone H3 and H4 [15,17], all show a clear drop in each of these histone signals upon induction. Also, our finding that as many as 50% of the promoter nucleosome sites register as naked prior to induction is consistent with other studies indicating a low nucleosome density at various yeast promoters, and it has been reported that, for many yeast genes, promoter nucleosomes turn over more rapidly than do ORF nucleosomes [37,38]. Taken together, these results are simply explained by the idea that promoter nucleosomes are often vacant prior to induction and increasingly so as induction proceeds. It is possible, but not directly demonstrated, that the relative absence of promoter nucleosomes prior to induction is determined by the intrinsic sequence of those nucleosome-forming sites. The fact that promoter nucleosomes must be removed for rapid activation of transcription indicates that even the relatively infrequent formation of these nucleosomes (compared, for example, to that observed for the ORFs) suffices to significantly compete with formation of the large, multicomponent transcription complex.

### The Assay

Our assay measures two aspects of nuclease protection conferred by a DNA bound molecule: the location of the bound molecule, the fraction of the population that bears it, and the degree of protection it imparts. Most DNA segments, as we have seen, yield biphasic digestion curves, and for segments bearing positioned nucleosomes, the ratio of the fast-digesting and slow-digesting fractions is very large, invariably close to a value of 200. Two unusual cases illustrate further how we can separate the degree of protection from the fractional occupancy. First, as we have seen, the molecule occupying 100% of the *UASg*'s in the population imparts a degree of protection some 10-fold less than that imparted by a nucleosome, and indeed this property is one of the indications that it is not a nucleosome. Second, we have introduced into the *GAL* locus a sequence predicted to have a high propensity to form a nucleosome ([39] and E. Segal and J. Widom, personal correspondence). This segment indeed forms a nucleosome (as measured in a ChIP experiment). In this case, 100% of the population is occupied, and the degree of protection is just that found for the typical nucleosome (i.e., some 200-fold) (X. Wang, G. O. Bryant, and M. Ptashne, unpublished data).

Our results do not exclude the possibility that nucleosomes, even positioned nucleosomes, can have some small degree of mobility along the DNA. In fact, the HS sites that lie between positioned nucleosomes are, as we have noted, naked to about the 90% level prior to induction, a value that decreases still further upon induction. Thus, perhaps, even the positioned nucleosomes can vary a few base pairs in their exact location in different members of the population.

## Materials and Methods

### Strains and growth conditions.

Strains, both wild type and deletions (except *gal4*Δ and FLAG-tagged H2B [40]), were derived from BY4741 (*MATa his3*Δ*1 leu2*Δ*0 met15*Δ*0 ura3*Δ*0*) obtained from EUROSCARF (European *Saccharomyces Cerevisiae* Archive for Functional Analysis). Additional strains used were CY1069 (*snf2Δ*) and its corresponding wild type [20]. We also added a wild-type *SNF2* expression plasmid, pM4724 [41], to both the BY4741 *snf2Δ* derivative and to CY1069. For all experiments reported, cells were grown exponentially for at least 16 h at 30 °C prior to harvesting in synthetic complete medium (SC) or, in the case of *snf2*Δ and its wild-type control, in yeast extract peptone medium (YP). All sugars were added at a final concentration of 2% unless otherwise indicated. For the galactose induction experiments, prewarmed and aerated galactose was added directly to the media. In cases in which harvested time points where less then 5 min apart, medium containing 4% galactose was added at a one-to-one ratio to the growing cells. For the glucose repression experiments, exponentially growing cells were precipitated, washed with the original medium, and then added to prewarmed and aerated glucose containing medium under conditions in which the original medium was diluted greater then 100-fold. For all experiments, cells were harvested at an optical density at 600 nm (OD_600_) between 0.5–0.9 by fixing the cells with freshly diluted formaldehyde at a final concentration at 0.5% for 1 to 5 min. The fixing reaction was stopped by addition of glycine at a final concentration of 0.125 M.

### Real-time PCR assay.

DNA and cDNA were quantitated by real-time quantitative polymerase chain reaction (real-time PCR). A 2× reaction buffer: 20 mM Tris-HCl (pH 8.3), 13 mM MgCl_2_, 100 mM KCl, 400 μM dNTPs, 4% DMSO, 2× SYBR Green I (Molecular Probes), 0.01% Tween 20, 0.01% NP40, 1–4 ng/μl of each oligo primer, and 0.025–0.1 U/μl of Taq polymerase (Roche) was mixed with an equal volume of DNA being quantitated, resulting in a total reaction volume of 5 μl. The time that the oligo primers are mixed with the 2× reaction buffer is limited to less then 10 min before it is mixed with the DNA sample to limit primer dimer formation. A typical real-time PCR reaction measured 80 unknown samples plus 16 known samples (two standard curves) in quadruplicate, i.e., 96 samples transferred into four different positions of a 384-well plate. Seven of the eight samples within a standard curve consisted of 3.33-fold dilution series of yeast chromosomal DNA. The final sample of the standard curve contained no DNA. All real-time PCR reactions were performed on the Light Cycle 480/384 from Roche. Reactions were run for 40 to 50 cycles (depending on the primer pair) at 95 °C for 4 s, 59 °C for 26 s, and 72 °C for 4 s with the florescence of the SYBR Green being read at the 72 °C step. Since the specific activity of Taq polymerase varied considerable from lot to lot, care was taken to test by titration each batch of Taq to find its optimal concentration.

Outliers of quadruplicate measurements were eliminated if dropping one of the four measurement reduced the standard deviation by greater then 2-fold and the original standard deviation was above the 50th percentile for the plate. An average quadruplicate measurement was eliminated if it was not greater then 2-fold above the measured value of the no DNA control. The DNA concentration was then determined by comparing real-time PCR measured values to a linear fit of the known chromosomal concentrations.

### mRNA analysis.

mRNA was isolated from 10 ml (growing volume) of cells by a modified version of the hot acidic phenol technique [42] in which the 65 °C incubation step was extended to 3 h to ensure that the formaldehyde crosslink was completely reversed. One twentieth of the isolated mRNA was reverse transcribed by AMV reverse transcriptase (Roche) as per the manufacturer's instructions. cDNA was quantitated by real-time PCR (see above) at *GAL1*, *GAL10*, *GAL7*, *GAL3*, and *GAL2* and for controls *HHF1*, *ACT1*, and *RBP11* (see Primer Pair List, Table S1). The three control genes were used to normalize the varying yields of cDNA from each sample.

### Chromatin immunoprecipitation assay.

ChIP assays were performed as described [1].

### Micrococcal nuclease assay.

Reaction conditions: 100–200 ml of a yeast cell culture were spun down and resuspended in 500 μl of FA lysis buffer without EDTA: 50 mM Hepes-KOH (pH 7.5), 140 mM NaCl, 1% Triton X-100, 0.1% sodium deoxycholate. The resuspended cells were sonicated twice for 10 s using a Branson Sonifier 250 equipped with a micro tip with the output set at 4. Cell debris was then spun down and the chromatin supernatant transferred to a new tube. A total of 26 μl was then distributed to 16 separate tubes, and 120 μl of FA lysis buffer without EDTA was added. To each tube, 10 μl of a micrococcal nuclease solution in H_2_O was added at a range of concentrations from 4 U to 0.000488 U in a 2-fold dilution series; two tubes had no nuclease. The reaction was started by adding 5.6 μl of 2 mM CaCl_2_ to each tube. The reactions were incubated for 1.5 h at 37 °C and stopped by the addition of 8.8 μl of 0.5 M EDTA each. Ten microliters of a solution containing 200 mM Tris (pH 7.4), 4 M NaCl, and 0.2 μl of Protease K (recombinant, Roche) were added to each tube, followed by incubation at 42 °C for 1 h and at 65 °C for at least 4 h. The DNA was purified using the QIAquick 96 PCR Purification Kit (Qiagen). DNA was typically quantitated at 16 or more positions (see Primer Pair List in Table S1) near the *GAL* genes (*GAL1*, *GAL10*, *GAL7*, *GAL2*, and *GAL3*, along with three positions within the *UASg*) and eight loci near the control genes *TUB2* and *PHO5*.

For purposes of discussion, the DNA from each level of micrococcal digestion will be referred to as a sample. All 16 samples from the same harvest digested at the range of micrococcal nuclease concentrations will be referred to as a group. The real-time PCR-measured value for each sample at each position (locus) will be referred to as the sample-locus value, and the group of values at each locus as group-locus values.

For each group-locus, the undigested DNA concentration was rescaled to one by dividing each sample-locus value of a group-locus by the average of the undigested sample-locus values. To compensate for the varying yields of DNA for each sample within a group (e.g., differing efficiencies of DNA recovery in the DNA purification step), the rescaled values for each group-locus measured at the *UASg* was fit to the one-state decay function e^−(*k*MN)^, where MN is the concentration of micrococcal nuclease and *k* is the adjustable parameter representing the rate of digestion. Each rescaled sample-locus is normalized by dividing it by the average of the sample-locus value (at the *UASg*) divided by its calculated one-step curve fit value. The normalized rescaled values for each group-locus is then fit to the two-state decay function (1 − fr_2_) e^−(*k*_1_^ ^MN)^ + fr_2_ e^−(*k*_2_^ ^MN)^, where MN is the concentration of micrococcal nuclease, *k*_1_ is the adjustable parameter representing the rate of digestion of the unprotected DNA, *k*_2_ is the adjustable parameter representing the rate of digestion of the protected DNA, and fr_2_ is the adjustable parameter representing the fraction of the DNA that is protected. The curve is fit by adjusting all three parameters to minimize the sum of the squares of the differences between the two-state decay function and the normalized rescaled group-locus values, where *k*_1_ is at least 50-fold greater than *k*_2_, and *k*_1_ is no less than a cutoff value set for each group (this cutoff value is typically 10–30 times greater than the protection seen within the *UASg* for the group). Sample-locus outliers from this fit were eliminated if the absolute difference of the normalized rescaled sample-locus value compared to its respective curve was greater then five times the average absolute difference for the entire group. The curves were then fit again to the remaining data points as described above. The error for each adjustable parameter was calculated by incrementally adjusting the parameter away from its best fit while allowing the other two parameters to adjust to their minimum until the sum of the squares of the differences increased by greater than 10%.

A slight systematic variation in fr_2_ values was seen at the control loci (the variation was less then 15%). To correct for this, the average value for each control was assumed to be its true value. For each group, the measured fr_2_ values at the control loci were plotted against their average values, and a curve fit was then performed on this plot. The curve used was the line segments defined by the points ((0,0), (*x*,*y*)) and ((*x*,*y*), (1,1)), where the *x*-axis is the measured fr_2_ control values and the *y*-axis is the average control values. A least-squares fit was performed by adjusting *x* and *y* under conditions in which the slope of either line segment was between 0.5 and 2. The fit curve was then used to rescale all fr_2_ measurements from the group.

## Supporting Information

Figure S1 Protection Pattern at the *Gal1,10* Locus Before and 20 min After Induction (A) This redrawing of the data of Figure 2B shows the protection pattern and nucleosome array for cells grown in raffinose. Here, each bar represents the position of an approximately 60-bp amplicon, and the height of each depicts the percent occupied as described in the text. That is, the height of each bar corresponds to the fraction of the corresponding horizontal bar of Figure 2B that is green.
(B) The same as for (A) except that 20 min prior to harvesting the cells, galactose (2%) was added to cells growing in raffinose. This figure shows that after induction, despite the presence of the transcriptional machinery as revealed by ChIP analysis (see Figure 4C and [1]), there is no significant protection of the promoter over and around the TATA box.
(300 KB PDF)

Figure S2 Improved Resolution of a “High-Resolution” ChIP of Flag-Tagged Histone H2B The thick line is identical to that of Figure 2E showing nucleosome positioning around the *UASg* for cells growing in raffinose. These data were generated as a “high-resolution” ChIP. That is, after cross-linking and sonication, the chromatin was treated lightly with micrococcal nuclease before immunoprecipitation. The experiment that generated the data represented by the thin dotted line, in contrast, omitted this nuclease step. Sonication alone generates fragments of about 500 bp, and the addition of the nuclease step evidently decreases this fragment length sufficiently to dramatically improve resolution.
(228 KB PDF)

Figure S3 Protection at the *UASg* Compared with That Effected by a Nucleosome Shown are nuclease digestions of two purified DNA segments, one from the *UASg* (A) and one from the *GAL1* ORF (B) (blue lines). Also shown are digestions of these segments as found in cross-linked chromatin (magenta lines). The curves in (B) are identical to those shown in Figure 1B of the text. These curves, along with numerical rates of digestion (see legend to Figure 1 in text), reveal that the molecule inferred to be bound to the *UASg* (A) confers less protection than does a nucleosome (B).
(306 KB PDF)

Figure S4 Nucleosome Occupancy of a Promoter versus ORFs Each dot represents an approximately 60-bp fragment found in the *GAL1,10* promoter (excluding the *UASg*) or at one or another position in the *GAL1*, *PHO5*, or*TUB2* ORFs. Cells were grown in the absence of galactose and the presence of phosphate, and so *GAL1* and *PHO5* are off. The highest points of occupancy in the *GAL1,10* promoter correspond to the centers of the positioned nucleosomes flanking the *UASg*. The nucleosomes in the ORFs are not well positioned. The figure shows that, at many positions in the ORFs, the average protection is higher than in the *GAL1,10* promoter. Note the values below 20% found in the promoter region—these are HS sites, and none are found in the ORFs.
(255 KB PDF)

Figure S5 Deletion of *SNF2* Significantly Delays Induction of the *GAL1* Gene The depicted experiment used two putative *SNF2*-deleted strains (BY4741 *ΔSNF2*—obtained from EUROSCARF, and CY1069—kindly provided by Craig Peterson). Each strain was transformed with a plasmid expressing *SNF2* (pM4724) to produce two pairs of putative isogenic *SNF2* ^+/−^ strains. The figure shows that for each pair, the strain expressing SNF2 induced significantly more quickly, as assayed by production of *GAL1* mRNA, than did the strain lacking *SNF2*.
(354 KB PDF)

Figure S6 Presence of Gal4 at the *UASg* at Early Times Following the Onset of Glucose Repression Two ChIP experiments are shown using an antibody to Gal4.
(A) Cells were pregrown in galactose and raffinose, and at time zero transferred to glucose.
(B) Cells were pregrown in galactose and raffinose, and at time zero transferred to medium containing three sugars: glucose, galactose, and raffinose (see also text Figure 4C).
(322 KB PDF)

Table S1 Real-Time PCR Primer Pair List The table lists each primer pair used for this paper with its: name, size, midpoint (relative to the ATG for promoter and ORF primers and relative to the stop site for terminator primers), and the sequence of each oligo.
(217 KB PDF)

## Abbreviations

ChIP: chromatin immunoprecipitation
HS: hypersensitive
*UASg,*: upstream activating sequence, galactose
