# Supplementary figures and images for: Activator Control of Nucleosome Occupancy in Activation and Repression of Transcription
Source: PLoS Biol. 2008 Dec 23;6(12):e317. doi: 10.1371/journal.pbio.0060317 (PMC2605919; doi:10.1371/journal.pbio.0060317)

A

GAL4 uninduced

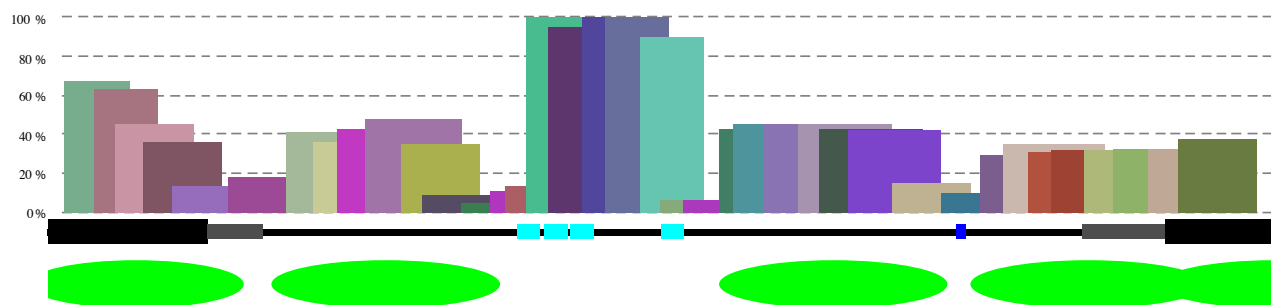

B

GAL4 induced

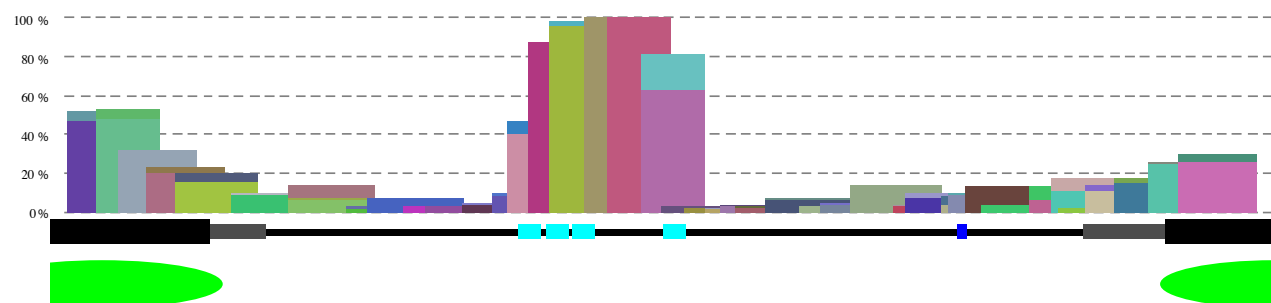

Supplement: Figure S1 — (A) This redrawing of the data of Figure 2B shows the protection pattern and nucleosome array for cells grown in raffinose. Here, each bar represents the position of an approximately 60-bp amplicon, and the height of each depicts the percent occupied as described in the text. That is, the height of each bar corresponds to the fraction of the corresponding horizontal bar of Figure 2B that is green. (B) The same as for (A) except that 20 min prior to harvesting the cells, galactose (2%) was added to cells growing in raffinose. This figure shows that after induction, despite the presence of the transcriptional machinery as revealed by ChIP analysis (see Figure 4C and [1]), there is no significant protection of the promoter over and around the TATA box. (300 KB PDF) [file pbio.0060317.sg001.pdf]

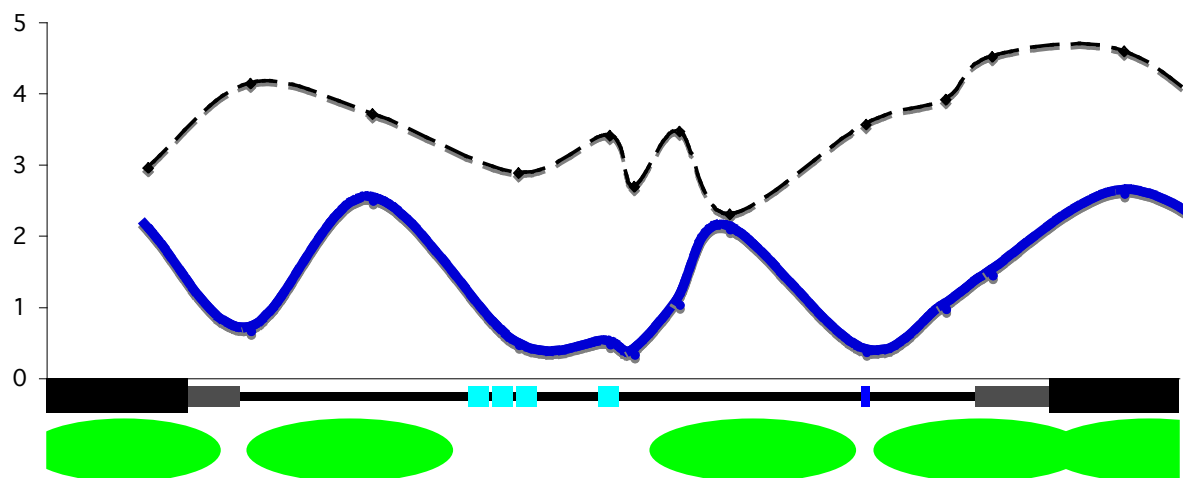

Supplement: Figure S2 — The thick line is identical to that of Figure 2E showing nucleosome positioning around the UASg for cells growing in raffinose. These data were generated as a “high-resolution” ChIP. That is, after cross-linking and sonication, the chromatin was treated lightly with micrococcal nuclease before immunoprecipitation. The experiment that generated the data represented by the thin dotted line, in contrast, omitted this nuclease step. Sonication alone generates fragments of about 500 bp, and the addition of the nuclease step evidently decreases this fragment length sufficiently to dramatically improve resolution. (228 KB PDF) [file pbio.0060317.sg002.pdf]

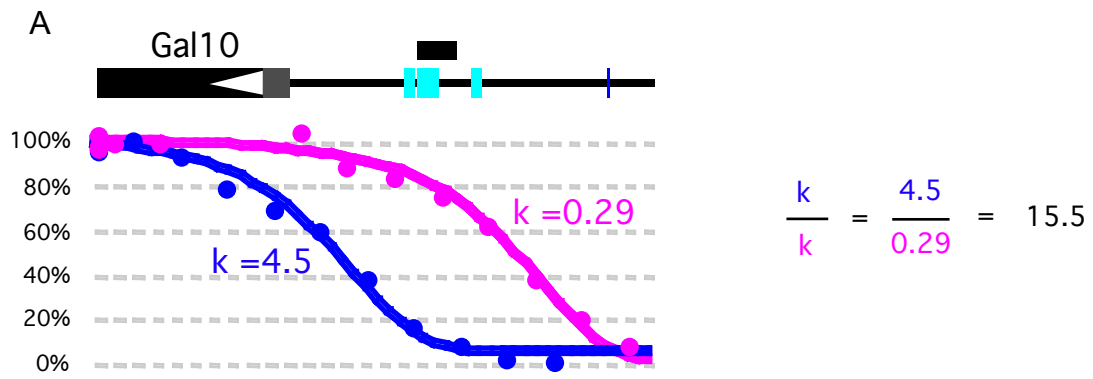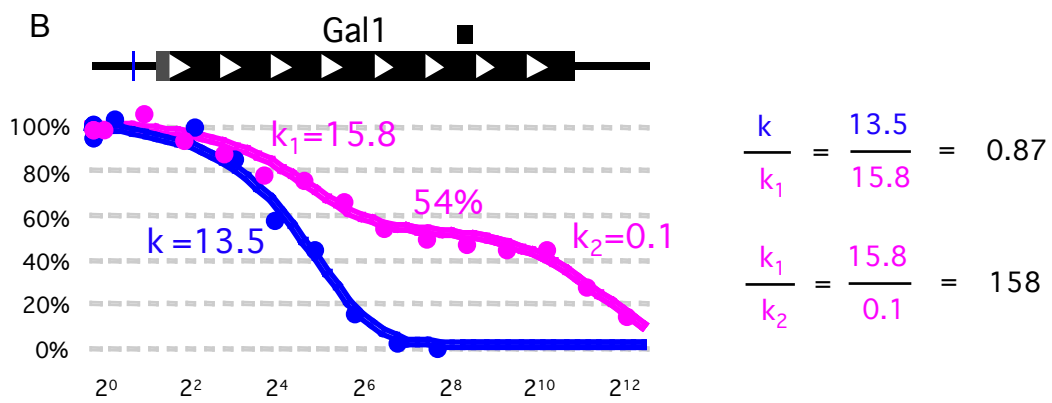

Supplement: Figure S3 — Shown are nuclease digestions of two purified DNA segments, one from the UASg (A) and one from the GAL1 ORF (B) (blue lines). Also shown are digestions of these segments as found in cross-linked chromatin (magenta lines). The curves in (B) are identical to those shown in Figure 1B of the text. These curves, along with numerical rates of digestion (see legend to Figure 1 in text), reveal that the molecule inferred to be bound to the UASg (A) confers less protection than does a nucleosome (B). (306 KB PDF) [file pbio.0060317.sg003.pdf]

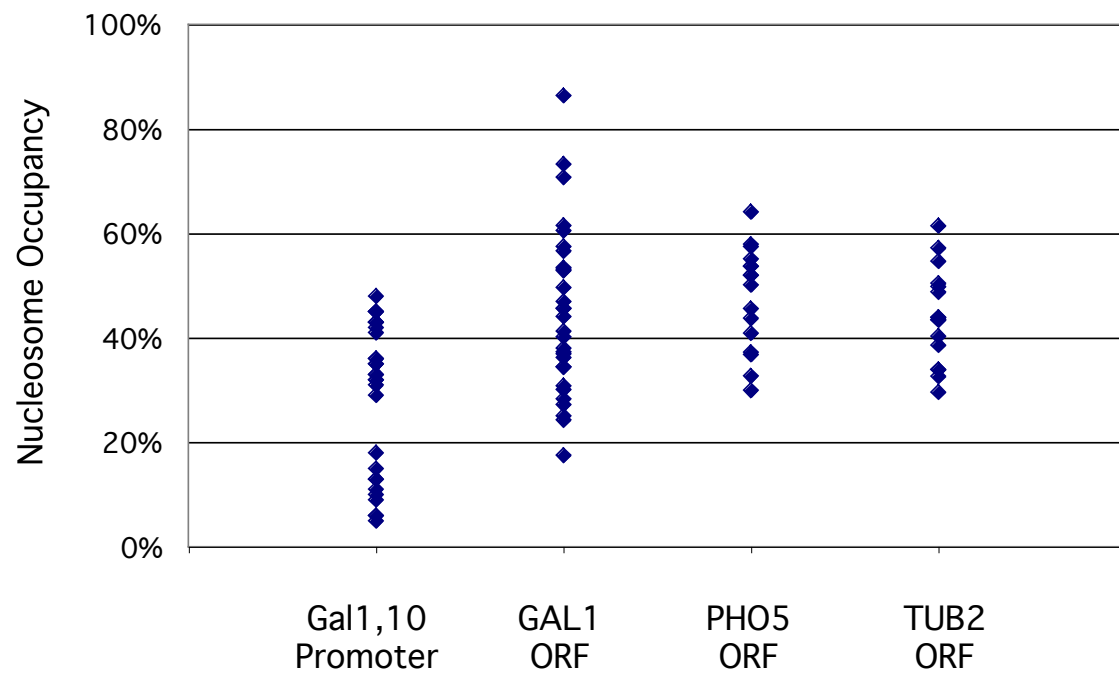

Supplement: Figure S4 — Each dot represents an approximately 60-bp fragment found in the GAL1,10 promoter (excluding the UASg) or at one or another position in the GAL1, PHO5, orTUB2 ORFs. Cells were grown in the absence of galactose and the presence of phosphate, and so GAL1 and PHO5 are off. The highest points of occupancy in the GAL1,10 promoter correspond to the centers of the positioned nucleosomes flanking the UASg. The nucleosomes in the ORFs are not well positioned. The figure shows that, at many positions in the ORFs, the average protection is higher than in the GAL1,10 promoter. Note the values below 20% found in the promoter region—these are HS sites, and none are found in the ORFs. (255 KB PDF) [file pbio.0060317.sg004.pdf]

A

BY4741 ( $\Delta$ snf2) &  
BY4741 ( $\Delta$ snf2) pM4724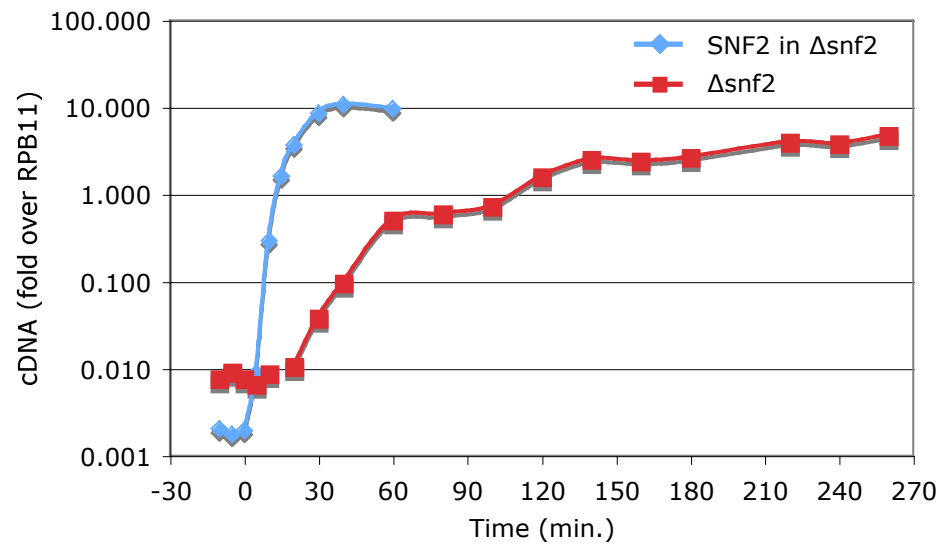

B

CY1069 &  
CY1069 pM4724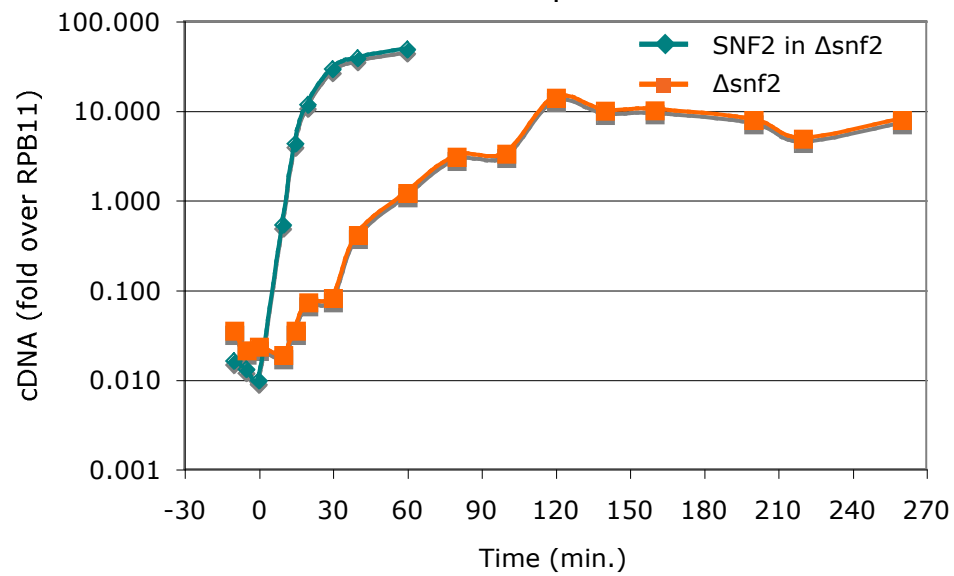

Supplement: Figure S5 — The depicted experiment used two putative SNF2-deleted strains (BY4741 ΔSNF2—obtained from EUROSCARF, and CY1069—kindly provided by Craig Peterson). Each strain was transformed with a plasmid expressing SNF2 (pM4724) to produce two pairs of putative isogenic SNF2 +/− strains. The figure shows that for each pair, the strain expressing SNF2 induced significantly more quickly, as assayed by production of GAL1 mRNA, than did the strain lacking SNF2. (354 KB PDF) [file pbio.0060317.sg005.pdf]

A

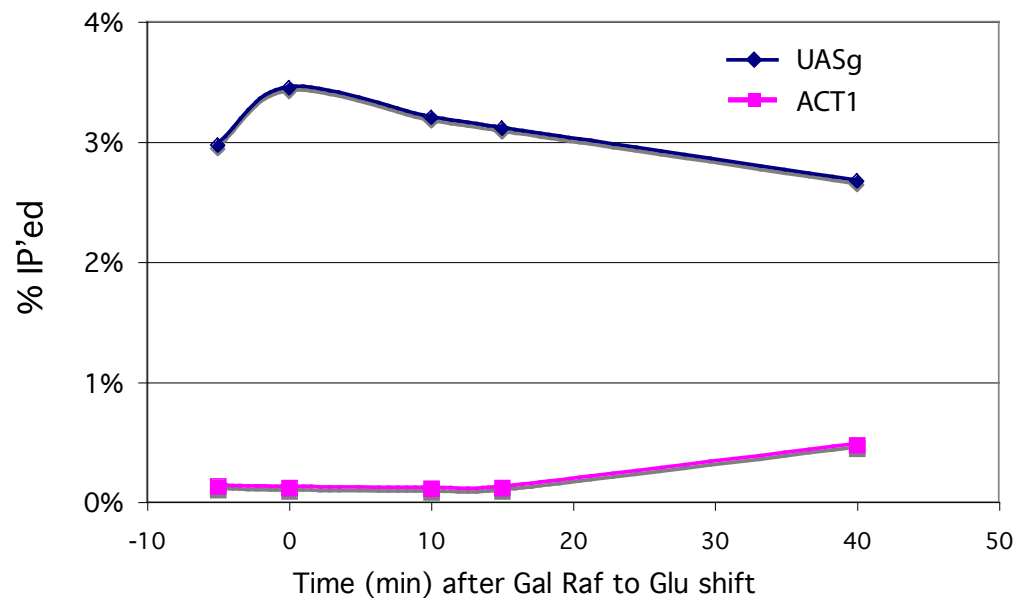

B

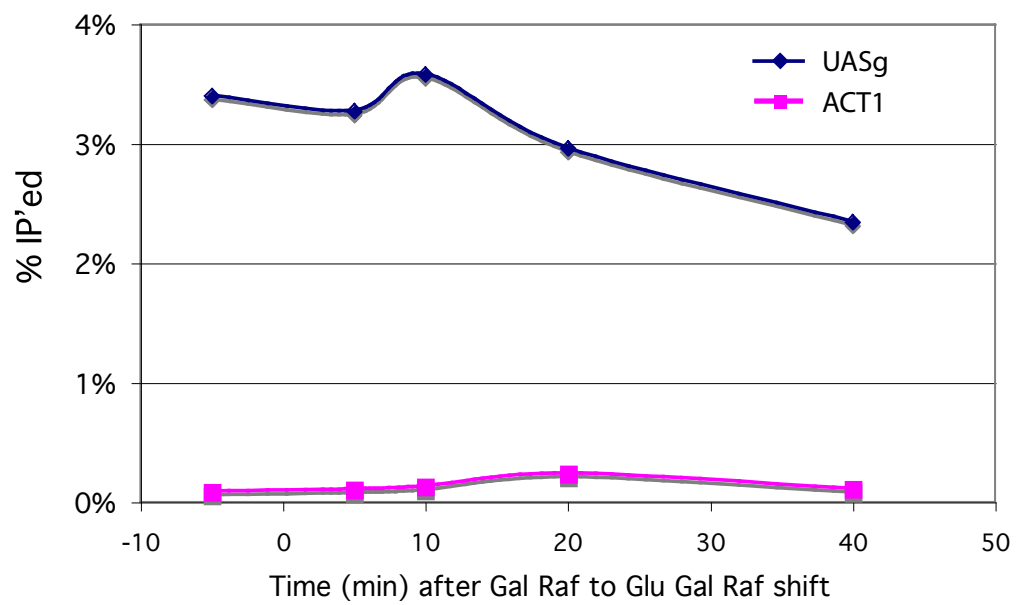

Supplement: Figure S6 — Two ChIP experiments are shown using an antibody to Gal4. (A) Cells were pregrown in galactose and raffinose, and at time zero transferred to glucose. (B) Cells were pregrown in galactose and raffinose, and at time zero transferred to medium containing three sugars: glucose, galactose, and raffinose (see also text Figure 4C). (322 KB PDF) [file pbio.0060317.sg006.pdf]
