# Supplementary material for: Activator Control of Nucleosome Occupancy in Activation and Repression of Transcription
Source: PLoS Biol. 2008 Dec 23;6(12):e317. doi: 10.1371/journal.pbio.0060317 (PMC2605919; doi:10.1371/journal.pbio.0060317)
Supplement: Table S1 — The table lists each primer pair used for this paper with its: name, size, midpoint (relative to the ATG for promoter and ORF primers and relative to the stop site for terminator primers), and the sequence of each oligo. (217 KB PDF) [file pbio.0060317.st001.pdf]

Primer Pair Name	Size	Location			Sequence
Hhf1orf01	45	Hhf1	ORF	206.0	TTCTTGGAATCCGTCATCAGAG<br>GTGTTCGGTGTAGGTAACAGAGT
Gal7MN1037	61	Gal7	Terminator	170.0	TTTTTTTTTTTTTATGGAAAGGACCAC<br>ATCTGATTCTTAATGCTATTCTAGTTA
Gal7MN1058	61	Gal7	ORF	1050.0	AATCTGACCATCTAAATTTCTTAGTTT<br>TCAAAGAGATTTAACTTCGGAACAA
Gal7MN1074	61	Gal7	ORF	890.0	TCATCACCAGTCGCATTCAAAG<br>AAACGAGTTTCCCATACTCAATG
Gal7MN1002	81	Gal7	ORF	10.0	TAAAAAAAAACAGTTGAATATTCCCTC<br>TTGTAACGTCTATGGGAATGGC
Gal7MN1009	61	Gal7	Promoter	-61.0	AAAGATATAAAAGCAGGTCGGAAAT<br>ATCATGTTGATGCTCTGCATAATAA
GalMN2221	61	Gal10	ORF	550.0	TAAAATAACGCAAGATAGCAAACTTC<br>TTGAATGATCTTTACAATAGCGACA
GalMN2225	61	Gal10	ORF	510.0	GCTATTGTAAAGATCATTCAAGATATT<br>TAATCCGTATGGTCATACGAAATAC
GalMN2229	61	Gal10	ORF	470.0	TTCGTATGACCATACGGATTAGT<br>ATATGATTCCTATCCCAGAAGAATG
GalMN2233	61	Gal10	ORF	430.0	CTTCTGGGATAGGAATCATATTTG<br>TCTTCTGCTACTGTCTATGGTG
GalMN2237	61	Gal10	ORF	390.0	ACCATAGACAGTAGCAGAAGATG<br>GTTAATGCAACAATACAACGTTTCC
GalMN2241	61	Gal10	ORF	350.0	ACGTTGTATTGTTGCATTAACTCTA<br>ACTATCACAATAACATTTTGGGAAC
GalMN2245	61	Gal10	ORF	310.0	CCAAAATGTTATTGTGATAGTATCTC<br>TTAAAGGCTGTAGGTGAATCTAC
GalMN2249	61	Gal10	ORF	270.0	AGATTCACCTACAGCCTTTAAAC<br>CAAAGAATATAAAATTGATTCGGTAATTC
GalMN2253	61	Gal10	ORF	230.0	GAATCAATTTTATATTCTTTGAAAACCTT<br>AGGTTGATTTGTGTGACCGAAAA
GalMN2257	61	Gal10	ORF	190.0	TTCGGTCACACAAATCAACCTC<br>AGGTTAGAGGTCTTGACCAAG
GalMN2261	61	Gal10	ORF	150.0	CTTGGTCAAGACCTCTAACCT<br>TGCTGATAACCTGTCGAATTCAA
GalMN2265	61	Gal10	ORF	110.0	GAATTCGACAGGTTATCAGCAAC<br>CTGTGGTAGAGCTAATTGAGAAT
GalMN1579	46	Gal10	ORF	77.5	TCTCAATTAGCTCTACCACAGT<br>GGTGCTGGATACATTGGTTCA
GalMN1591	46	Gal10	ORF	57.5	GTGTGTGAACCAATGTATCCAG<br>AAATTGTTTTGGTTACAGGTGGTG
GalMN1602	56	Gal10	ORF	37.5	TATCCAGCACCACCTGTAACC<br>CAGTTACAAAGTGAAAGTACTTCTAA
GalMN1612	56	Gal10	ORF	17.5	CAAAACAATTTTAGAAGTACTTTCACT<br>ATTCAATATAAATGACAGCTCAGTTA
GalMN1623	59	Gal10	Promoter	-5.0	TTTCACTTTGTAACTGAGCTGTCA<br>AAAAAAAGTAAGAATTTTTGAAAATTCAATATA

Primer Pair Name	Size	Location		Sequence
GalMN1643	61	Gal10 Promoter	-46.0	AAAAATTCTTACTTTTTTTTTGGATGGAC<br>TGCCATGTAATATGATTATTAAACTTCT
GalMN1664	61	Gal10 Promoter	-86.0	TAATAATCATATTACATGGCATTACCA<br>ATATAAGTAAGATTAGATATGGATATGTA
GalMN1678	63	Gal10 Promoter	-105.0	GCATTACCACCATATACATATCCA<br>GCTCTTTACATTTCCACAACATATAA
GalMN1687	66	Gal10 Promoter	-123.5	ATATCCATATCTAATCTTACTTATATGTT<br>TTTTAGGCTAAGATAATGGGGCT
GalMN1689	56	Gal10 Promoter	-123.5	CATATCTAATCTTACTTATATGTTGTG<br>GGCTAAGATAATGGGGCTCTTT
GalMN1699	69	Gal10 Promoter	-145.0	TTATATGTTGTGGAAATGTAAAGAGC<br>TGAAAGTTCCAAAGAGAAGGTTTT
GalMN1713	56	Gal10 Promoter	-163.5	CCCCATTATCTTAGCCTAAAAAAAC<br>TAAGCGTATTACTGAAAGTTCCAAA
GalMN1179	71	Gal10 Promoter	-186.0	CTAAAAAAACCTTCTCTTTGGAACTTT<br>AATCCGTACTTCAATATAGCAATGA
GalMN1726	56	Gal1 Promoter	-463.5	CAGTAATACGCTTAACTGCTCAT<br>TCGGCGGCTTCTAATCCGT
GalMN1736	56	Gal1 Promoter	-443.5	CATTGCTATATTGAAGTACGGATTA<br>GTCGGAGGGCTGTCGCC
GalMN1749	41	Gal1 Promoter	-426.0	GAAGCCGCCGAGCGGG<br>AGAGTCTTCCGTCGGAGGG
GalMN1752	56	Gal1 Promoter	-413.5	CGCCGAGCGGGCGACAGC<br>AAGACGAGGACGCACGGAG
GalMN1190	61	Gal1 Promoter	-401.0	GCGACAGCCCTCCGACGG<br>GGAACGCGACCGGTGAAG
GalMN1768	41	Gal1 Promoter	-386.0	TCCTCCGTGCGTCCTCG<br>TTTCAGGAACGCGACCGGT
GalMN1775	46	Gal1 Promoter	-368.5	CGTCTTCACCGGTCGCGT<br>CGGCGCGAGGCACATCT
GalMN1792	46	Gal1 Promoter	-343.5	ACGCAGATGTGCCTCGCG<br>AGAATCTTTATTGTTCGGAGCAGT
GalMN1800	58	Gal1 Promoter	-324.5	TCGCGCCGCACTGCTCCG<br>TCATAACCATAAAAGCTAGTATTGTAG
GalMN1814	66	Gal1 Promoter	-303.5	GAACAATAAAGATTCTACAATACTAGC<br>CCAGGTTACTGCCAATTTTTCC
GalMN1823	66	Gal1 Promoter	-288.5	TACAATACTAGCTTTTATGGTTATGAA<br>TGAAGGTTTGTGGGGCCAG
GalMN1496	56	Gal1 Promoter	-283.5	GCTTTTATGGTTATGAAGAGGAAAAAT<br>TGAAGGTTTGTGGGGCCAG
GalMN1366	71	Gal1 Promoter	-266.0	TTATGAAGAGGAAAAATTGGCAGTA<br>TGGTTGTTAATTTGATTCGTTAATTTG
GalMN1211	62	Gal1 Promoter	-249.5	AGTAACCTGGCCCCACAAAC<br>ATTATCATCCTATGGTTGTTAATTTGAT
Gal1MN51	75	Gal1 Promoter	-243.0	AGTAACCTGGCCCCACAAAC<br>AAAAACTAATCGCATTATCATCCT

Primer Pair Name	Size	Location	Sequence
GalMN1401	66	Gal1 Promoter -223.5 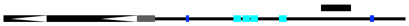	AAATTAACGAATCAAATTAACAACCATAG<br>CCAGAAATAAGGCTAAAAAACTAATC
GalMN1289	73	Gal1 Promoter -205.0 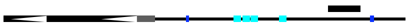	ATTAACAACCATAGGATGATAATGC<br>ATCGCTTCGCTGATTAATTACCC
GalMN1528	66	Gal1 Promoter -188.5 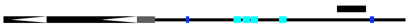	AATGCGATTAGTTTTTAGCCTTATTT<br>TAGATCAAAAATCATCGCTTCGC
GalMN1533	56	Gal1 Promoter -163.5 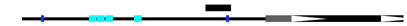	GGGTAATTAATCAGCGAAGCGA<br>TCCATTTATATATCTGTTAATAGATCAAA
GalMN1441	76	Gal1 Promoter -143.5 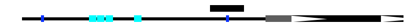	TCAGCGAAGCGATGATTTTTGAT<br>AAGTATTAGTTAAAGTGGTTATGCAG
GalMN1450	77	Gal1 Promoter -118.0 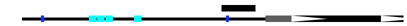	ATTAACAGATATATAAATGGAAAAGCTG<br>GAAGTAATACAAACTGAAAATGTTGAA
GalMN1349	91	Gal1 Promoter -106.0 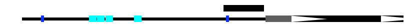	CAGATATATAAATGGAAAAGCTGCATA<br>TTTTATGACATTTGAATAAGAAGTAATAC
GalMN1350	89	Gal1 Promoter -85.0 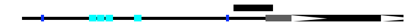	GCATAACCACTTTAACTAATACTTTCA<br>TAACAATTTTTTGTTGATACTTTTATGACA
GalMN1860	65	Gal1 Promoter -78.0 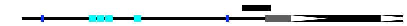	TACTTTCAACATTTTCAGTTTGTATTAC<br>ATTTTTTGTTGATACTTTTATGACATTTG
GalMN1233	73	Gal1 Promoter -77.0 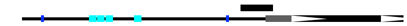	TAATACTTTCAACATTTTCAGTTTGTATT<br>TAACAATTTTTTGTTGATACTTTTATGACA
GalMN1868	70	Gal1 Promoter -60.5 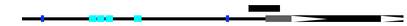	CAGTTTGTATTACTTCTTATTCAAATG<br>AGTATAGAGGTATATTAACAATTTTTTGT
GalMN1876	64	Gal1 Promoter -47.5 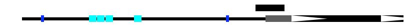	TTATTCAAATGTCATAAAAGTATCAACAA<br>TTGACGTTAAAGTATAGAGGTATATTA
GalMN1546	64	Gal1 Promoter -43.5 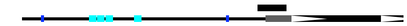	TCAAATGTCATAAAAGTATCAACAAAA<br>CTCCTTGACGTTAAAGTATAGAGGTAT
GalMN1887	66	Gal1 Promoter -23.5 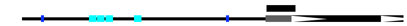	CAACAAAAAATTGTTAATATACCTCTATA<br>ATTTAGTCATTATAGTTTTTTCTCCTTG
GalMN1459	65	Gal1 Promoter -3.0 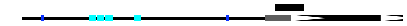	CCTCTATACTTTAACGTCAAGGAGAA<br>AATCACTTCTTCTGAATGAGATTTAGT
GalMN1901	61	Gal1 ORF 20.0 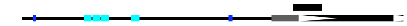	AAAAAACTATAATGACTAAATCTCATTCA<br>CTAGAATTGAACTCAGGTACAATCA
GalMN1914	56	Gal1 ORF 37.5 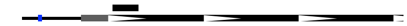	TCTCATTCAGAAGAAGTGATTGTAC<br>GGTAATTCCTTTGCGCTAGAATT
GalMN1928	51	Gal1 ORF 60.0 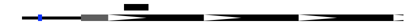	CTGAGTTCAATTCTAGCGCAAAG<br>ACTTTTCGGCCAATGGTCTTG
GalMN2049	46	Gal1 ORF 77.5 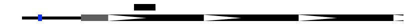	AAGGAATTACCAAGACCATTGGC<br>TAATTATGCTCGGGCACTTTTCG
GalMN2055	56	Gal1 ORF 92.5 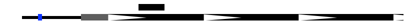	CAAGACCATTGGCCGAAAAGT<br>ATAAGCGCTTATAAATTTCTTAATTATGC
GalMN2062	61	Gal1 ORF 120.0 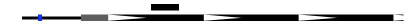	GAGCATAATTAAGAAATTTATAAGCGC<br>CGATCTAGCAACAAAATCCGGT
GalMN2065	51	Gal1 ORF 135.0 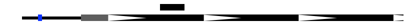	TAAGCGCTTATGATGCTAAACCG<br>CTCTACCAGGCGATCTAGC
GalMN2069	51	Gal1 ORF 155.0 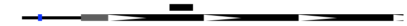	CCGGATTTTGTTGCTAGATCG<br>ATGTTCACCAATTAGATTGACTCTA

Primer Pair Name	Size	Location	Sequence
GalMN2075	51	Gal1 ORF 175.0	GCCTGGTAGAGTCAATCTAATTG<br>GAGAAGTCACAATAATCAATATGTTC
GalMN2079	51	Gal1 ORF 195.0	TTGGTGAACATATTGATTATTGTGAC<br>CAATAGCTAAAGGTAAAACCGAG
GalMN2085	51	Gal1 ORF 215.0	TGTGACTTCTCGGTTTTACCTTT<br>GGCGCAAAGCATATCAAAATCAA
GalMN2089	47	Gal1 ORF 237.0	GCTATTGATTTTGATATGCTTTGC<br>TTCTCGTTCAAAACTTTGACGGC
GalMN2092	57	Gal1 ORF 242.0	GCTATTGATTTTGATATGCTTTGC<br>GGATGGATTTTTCTCGTTCAAAAC
GalMN2095	51	Gal1 ORF 255.0	TGCTTTGCGCCGTCAAAGTTT<br>TTAAGGTAATGGATGGATTTTTCTC
GalMN2102	53	Gal1 ORF 276.0	TTGAACGAGAAAAATCCATCCATTA<br>GCAAATTTGGGATCAGCATT
GalMN2108	61	Gal1 ORF 300.0	CATTACCTTAATAAATGCTGATCCC<br>GTCCAACGGCAAATCGAACT
GalMN2112	51	Gal1 ORF 315.0	ATCCCAAATTTGCTCAAAGGAAG<br>CATAAGAACCGTCCAACGGC
GalMN2111	49	Gal1 ORF 316.0	CCCAAATTTGCTCAAAGGAA<br>CATAAGAACCGTCCAACGGC
GalMN2117	51	Gal1 ORF 335.0	AAGTTCGATTTGCCGTTGGAC<br>CACAGAAGGATCAATTGTGACAT
GalMN2121	51	Gal1 ORF 355.0	CGGTTCTTATGTCACAATTGATC<br>AAGTAATTAGACCAGTCCGACAC
GalMN1570	50	Gal1 ORF 374.5	ATCCTTCTGTGTCGGACTGG<br>AACATGGAGACCACATTTAAAGTAA
GalMN2132	61	Gal1 ORF 390.0	GTCGGACTGGTCTAATTACTTTA<br>TTTCTTTAGAAAAGAGTGAGCAACA
GalMN2140	61	Gal1 ORF 420.0	TCTCCATGTTGCTCACTCTTTTC<br>AGGAGCACTGGCAAACCTTTC
GalMN2142	47	Gal1 ORF 437.0	AAAGAAACTTGCACCGGAAA<br>GCCCGGCCAGAGGAGCAC
GalMN2148	41	Gal1 ORF 460.0	CCAGTGCTCCTCTGGCCG<br>ACCCTCACAGAAGACTTGCAG
GalMN2154	42	Gal1 ORF 479.5	GCTGCAAGTCTTCTGTGAGG<br>CCACTGCCAGTTGGTACATC
GalMN2165	51	Gal1 ORF 515.0	ACTGGCAGTGGATTGTCTTCT<br>TAAAGCAACGGCACAAATGAATG
GalMN2171	60	Gal1 ORF 550.5	TCATTTGTGCCGTTGCTTTA<br>ACATATGATAACCAGGGCCCA
GalMN2175	61	Gal1 ORF 590.0	TGGGCCCTGGTTATCATATGT<br>TGTTCTGCAACGACCGTAATAC
GalMN2179	61	Gal1 ORF 630.0	TATTACGGTCGTTGCAGAACATT<br>GGCAGCCTGATCCATACCG
GalMN2187	61	Gal1 ORF 710.0	ATCATGCTCTATACGTTGAGTTC<br>TGCGGAAATTTAAACGGAGTAGC

Primer Pair Name	Size	Location		Sequence
GalMN2191	61	Gal1 ORF	750.0	TACTCCGTTTAAATTTCCGCAATTA<br>GGTGTTCGCAATAACAAAGCTAA
GalMN2196	81	Gal1 ORF	800.0	AGCTTTGTTATTGCGAACACCC<br>TTCTACCACTCTTAAATTATAGTTGG
GalMN2199	61	Gal1 ORF	830.0	TTGAAACCGCCCCAACCAAC<br>ACATTTGCAGCTGTAGTGACTTC
GalMN2202	61	Gal1 ORF	870.0	AGTCACTACAGCTGCAAATGTTT<br>TTTTCCAGAAAGTAAAACAACACCG
GalMN2206	61	Gal1 ORF	910.0	GTTGTTTTACTTTCTGGAAAAGAAG<br>TGAAATCTCTTAGATTACCTTTATTCG
GalMN2210	61	Gal1 ORF	950.0	AAGGTAATCTAAGAGATTTCATGAAC<br>GGTGTGGAAATGTTGTGATATCTG
GalMN2214	61	Gal1 ORF	990.0	ATATCACAACATTTCCACACCCTG<br>TGTTAACCGTTCGATGCCGG
GalMN1939	51	Gal1 ORF	1025.0	TCCGGCATCGAACGGTTAAC<br>GAGAGACTCTTCAACTAGTACTA
GalMN1951	51	Gal1 ORF	1065.0	AAGAGTCTCTCGCCAATAAGAAA<br>GTGCGACATCGTCAACACTAA
GalMN1978	61	Gal1 ORF	1160.0	ACTTAACAACATCTCCAGTGAGA<br>ACATGCTTAGCCCTCTGATATAG
GalMN1985	61	Gal1 ORF	1170.0	ATCTCCAGTGAGATTTCAAGTCT<br>TTCAGAATACACATGCTTAGCCC
GalMN1988	75	Gal1 ORF	1197.0	TCTTAAAGCTATATCAGAGGGCT<br>TCATTAATTTCACAGCCTTCAAGA
Gal1MN89	58	Gal1 ORF	1234.5	AAGAGTCTTGAAGGCTGTGAAA<br>GTCTTCGTCGGCAGTAAAGC
GalMN1993	69	Gal1 ORF	1357.0	CGAATGTTCTTGTCCAGAGATTG<br>CGGGAACCATATGATCCATT
Pho5MN1001	61	Pho5 ORF	370.0	CGTCCGAGTTGGCAAAAGTG<br>GAGTTTTTCATCCGTGATGACG
Pho5MN1005	61	Pho5 ORF	330.0	GTCATCACGGATGAAAAACTCG<br>TCAATTCAACGGCTCATTGTCATT
Pho5MN1007	61	Pho5 ORF	310.0	CGTAATCATCGTTCAAGAATGACAA<br>AAGTTGAGCAATTACACTCGTCAAT
Pho5MN1009	61	Pho5 ORF	290.0	GACAATGAGCCGTTGAATTGAC<br>CTATCAAGAGTACATGGTATAAGTT
Pho5MN1011	61	Pho5 ORF	270.0	ACGAGTGTAATTGCTCAACTTATAC<br>TACTGTCAGTCTGGCTAAGACTA
Pho5MN1013	61	Pho5 ORF	250.0	TATACCATGTACTCTTGATAGTCTT<br>AGACATGGTGAAAGATACCCTAC
Pho5MN1015	61	Pho5 ORF	230.0	GTCTTAGCCAGACTGACAGTA<br>AGCAACTGCAAATGGTTGGTAG
Pho5MN1017	61	Pho5 ORF	210.0	AGGGTATCTTTCACCATGTCTAC<br>GCCTGAAGGTTGTGAAATGAAG
Pho5MN1019	61	Pho5 ORF	190.0	TACCAACCATTTGCAGTTGCTTC<br>TATGGTATTTCTCGTGATTTGCCT

Primer Pair Name	Size	Location	Sequence
Pho5MN1021	61	Pho5 ORF 170.0	TTCATTTCACAACCTTCAGGCAA<br>ACTACTCTTTCCCTGGCGAC
Pho5MN1023	61	Pho5 ORF 150.0	CAAATCACGAGAAATACCATAGTC<br>TTTGGGTGGTGCCGGACC
Pho5MN1026	61	Pho5 ORF 130.0	AGTCGCCAGGGAAAGAGTAG<br>CAAAAAGATATCTTCCCATTTTTGG
Pho5MN1028	61	Pho5 ORF 110.0	TATGGTCCGGCACCACCC<br>ATGTCGACAAGATTGGTACCCA
Pho5MN1030	61	Pho5 ORF 90.0	AAATGGGAAGATATCTTTTTGGGTA<br>TCCCTTAGGCAAACTAGCCG
Pho5MN1033	61	Pho5 ORF 70.0	GGGTACCAATCTTGTCGACAT<br>TTGGCCAATGCAGGTACCATT
Pho5MN1036	61	Pho5 ORF 50.0	TCGGCTAGTTTGCCTAAGGG<br>ATTCAATTTTAGCCGCTTCTTTGG
Pho5MN1040	61	Pho5 ORF 10.0	AAGAAGCGGCTAAAATTGAATAAACA<br>CAAGCAAATTCGAGATTACCAATGT
Pho5MN1042	61	Pho5 Promoter -11.0	TAAACAACAGATTTAAACATTGGTAATC<br>AAGAACAACAACAAATAGAGCAAGC
Pho5MN1044	61	Pho5 Promoter -31.0	TGGTAATCTCGAATTTGCTTGCT<br>GCTTCATCTCTCATGAGAATAAGAA
Pho5MN1045	81	Pho5 Promoter -41.0	TGGTAATCTCGAATTTGCTTGCT<br>GCTAAGTCGAGGTTAGTATGG
Pho5MN1046	61	Pho5 Promoter -51.0	GCTCTATTTGTTGTTGTTCTTATTC<br>GCTAAGTCGAGGTTAGTATGG
Pho5MN1048	61	Pho5 Promoter -71.0	TATTCTCATGAGAGATGAAGCCATA<br>TATATAAGCGCTGATGTTTTGCTAA
Pho5MN1050	61	Pho5 Promoter -91.0	CCATACTAACCTCGACTTAGCA<br>TGAATTGTCGAAATGAAACGTATATAA
Pho5MN1052	61	Pho5 Promoter -111.0	CAAAACATCAGCGCTTATATACGTT<br>GGGATAAGGGTAAACATCTTTGA
Pho5MN1054	61	Pho5 Promoter -131.0	ACGTTTCATTTCGACAATTCAAAGA<br>GGCAAGGCATATACCCATTTG
Pho5MN1056	61	Pho5 Promoter -151.0	AAAGATGTTTACCCTTATCCCAAAT<br>TCAAATTGGTCACCTTACTTGGC
Pho5MN1058	61	Pho5 Promoter -171.0	CAAATGGGTATATGCCTTGCCA<br>AGATCGCACATGCCAAATTATCAAA
Pho5MN1060	61	Pho5 Promoter -191.0	CAAGTAAGGTGACCAATTTGATAAT<br>TCTGGTCCCTGTTTTCGAAGA
Pho5MN1062	61	Pho5 Promoter -211.0	ATAATTTGGCATGTGCGATCTCT<br>GCACAGACTAAATTTATGATTCTGG
Pho5MN1064	61	Pho5 Promoter -231.0	TCTTCGAAAACAGGGACCAGAA<br>GGCACTCACACGTGGGAC
Pho5MN1066	61	Pho5 Promoter -251.0	AATCATAAATTTAGTCTGTGCTAGTC<br>AAATGAATCGATACAACCTTGGCA
Pho5MN1068	61	Pho5 Promoter -271.0	CTAGTCCCACGTGTGAGTGC<br>TAATTGAATAGGCAATCTCTAAATGAAT

Primer Pair Name	Size	Location	Sequence
Pho5MN1070	61	Pho5 Promoter -291.0	CAAGGTTGTATCGATTCATTTAGAG<br>GTAAAAGTGATTAAAAGAGTTAATTGAAT
Pho5MN1072	61	Pho5 Promoter -311.0	TAGAGATTGCCTATTCAATTAACTC<br>ACTGCACAATGCCAAAAAAAGTAAA
Pho5MN1076	61	Pho5 Promoter -351.0	CTTTTTTTGGCATTGTGCAGTTG<br>GAAATATATATTAAATTAGCACGTTTTCG
Pho5MN1080	61	Pho5p Promoter -391.0	TGCTAATTTAATATATATTTCTTTGTGCA<br>AGGTAAAAGGTTCATAGCGCTTT
Pho5MN1082	61	Pho5p Promoter -411.0	CTTTGTGCAGACAAAGAAAAAGC<br>CGTCTATAAACTTCAAACGAAGG
Pho5MN1084	61	Pho5p Promoter -431.0	AGCGCTATGAACCTTTTACCTTC<br>TTTCTCATGTAAGCGGACGTC
Pho5MN1086	61	Pho5p Promoter -451.0	TTCGTTTGAAGTTTATAGACGACG<br>TATGTGCGCTGCTTTAATGTTTTC
Pho5MN1088	61	Pho5p Promoter -471.0	GACGTCCGCTTACATGAGAAA<br>AACGTATTTGGAAGTCATCTTATGT
Pho5MN1090	61	Pho5p Promoter -491.0	AACATTAAAGCAGCGCACATAAG<br>CAAGAATGCGCAAATATGTCAAC
Pho5MN1092	61	Pho5p Promoter -511.0	AAGATGACTTCCAAATACGTTGACA<br>GATCCGAAAGTTGTATTCAACAAG
Pho5MN1094	61	Pho5p Promoter -531.0	TGACATATTTGCGCATTCTTGTTG<br>CCGGAGACCGGCATTACAA
Pho5MN1097	41	Pho5p Promoter -561.0	CCTTGTAATGCCGGTCTCC<br>CACGCTCTCTTTACAGGACG
Pho5MN1102	61	Pho5p Promoter -591.0	GCGTCCTGTAAAGAGAGCG<br>TTGACCTGATGTCAGTCCCC
Pho5MN1105	61	Pho5p Promoter -611.0	GCGACACGCCGCTATTAGC<br>AGCTCGCTACAATAATAATGTTGAC
Pho5MN1107	61	Pho5p Promoter -631.0	TGGGGACTGACATCAGGTC<br>TCTATTTACTGACCGAAAGTAGC
Pho5MN1109	61	Pho5p Promoter -651.0	ACATTATTATTGTAGCGAGCTACTT<br>GCTTGTATCTCTACATATGTTCTAT
Pho5MN1111	61	Pho5p Promoter -671.0	TACTTTCGGTCAGTAAATAGAACAT<br>GTCCGCTCCTTCTAATAATCG
Pho5MN1113	61	Pho5p Promoter -691.0	AACATATGTAGAGATACAAGCGATT<br>GTGACCCAACTTTGTTGTAGG
Pho5MN1115	61	Pho5p Promoter -711.0	CGATTATTAGAAGGAGCGGAC<br>AAGTAATAATTGCGAGAAACGTGAC
Pho5MN1119	61	Pho5p Promoter -751.0	CGTTTCTCGCAATTATTACTTGG<br>AAGTTACTACTGCACATTGGCAT
Pho5MN1121	61	Pho5p Promoter -771.0	TGGATGCCCTCCTAGCTAATG<br>TTTTTACACATCGGACTGATAAGTT
Pho5MN1123	61	Pho5p Promoter -791.0	GCCAATGTGCAGTAGTAACTTAT<br>TCGCAAAAAATTAGTTCTATTTTTTACAC
Pho5MN1125	61	Pho5p Promoter -811.0	TATCAGTCCGATGTGTAAAAAATAG<br>GATGGTTTTTGTCCATCTTTTCG

Primer Pair Name	Size	Location	Sequence
Pho5MN1127	61	Pho5p Promoter -831.0	AATAGAACTAATTTTTTGCGAAAAGATG<br>TAGTGAGAAAATTGACCAGAGATG
Pho5MN1129	61	Pho5p Promoter -851.0	AAAAGATGGACAAAAACCATCTCTG<br>CAAGAGACTCCGTCCCTCTT
Pho5MN1133	61	Pho5p Promoter -891.0	AAAGAGGGACGGAGTCTCTTG<br>GGGAAAATCAAAACATTCCCTGT
Pho5MN1135	61	Pho5p Promoter -911.0	GTTTTCTTCTATTAGTGCACAGG<br>AGAAGCGAAAGAAAAAAAAAGGGAAA
Pho5MN1137	61	Pho5p Promoter -931.0	AGGGAATGTTTTGATTTTCCCTTTT<br>TTTATTTTTACCACTGTTGAAGAAGC
Pho5MN1141	60	Pho5p Promoter -970.5	TTCAACAGTGGTAAAAATAAAAATTATTG<br>TAAATACAATGTTCCTTGGTTATCC
Gal3MN1009	61	Gal3 Promoter -391.0	ACCATCAGCCTCAAGTCGTC<br>ATAGATTGTTATCTATCTGCATGAAC
Gal3MN1038	61	Gal3 Promoter -131.0	GCAGTAAAATTTTTACTGAAACGTATA<br>AAGGTGTTGCCTCACTTGTCG
Gal3MN1051	61	Gal3 ORF 1470.0	GGTACGAAAAGCACTAATCGAAA<br>TTCATCTGTGAGATCCGGATATC
Act1Vid01	58	Act1 ORF 822.5	CGTTCCAATTTACGCTGGTT<br>GGCCAAATCGATTCTCAAAA
Tub2MN1102	61	Tub2p Promoter -571.0	GATCCCATGCGAACCACGG<br>TGCAGGTCAAGAACGTTTCCG
Tub2MN1104	61	Tub2p Promoter -551.0	AGTAAGATGAAGTGATAGTACGGA<br>AAGCTACAGATTTGGGACACTG
Tub2MN1106	61	Tub2p Promoter -531.0	CGGAAACGTTCTTGACCTGC<br>AACTGGACGGCAAGACTGTAA
Tub2MN1108	61	Tub2p Promoter -511.0	AGTGTCCCAAATCTGTAGCTTTA<br>CTTCAAGATTAAGACTGTAGAACTG
Tub2MN1110	61	Tub2p Promoter -491.0	TTACAGTCTTGCCGTCCAGTT<br>ATCTCCACAATTGGAGTGGAC
Tub2MN1112	61	Tub2p Promoter -471.0	TCTACAGTCTTAATCTTGAAGTCC<br>ACACATATACCAACGACTACATC
Tub2MN1114	61	Tub2p Promoter -451.0	GTCCACTCCAATTGTGGAGAT<br>ACTTTTGAGGTTTTCGGACGAC
Tub2MN1116	61	Tub2p Promoter -431.0	TGTAGTCGTTGGTATATGTGTCG<br>GGTGTCGGGAAGTCCTGTTT
Tub2MN1118	61	Tub2p Promoter -411.0	TCGTCCGAAAACCTCAAAAGTAA<br>TGCTGTTGATCGGGAATTCCG
Tub2MN1120	61	Tub2p Promoter -391.0	TAAACAGGACTTCCCGACACC<br>GTACGATTACCTGTTCAAACTGC
Tub2MN1001	61	Tub2 Promoter -371.0	CGGAATTCCCGATCAACAGC<br>AGCACTAATATGAATAGCGAGTAC
Tub2MN1003	61	Tub2 Promoter -351.0	AGTTTGAACAGGTAATCGTACTC<br>GAAATAAATAAAACAAGAAAAGCACTAAT
Tub2MN1005	61	Tub2 Promoter -331.0	CTCGCTATTCATATTAGTGCTTTTC<br>TTTACTGTTAGGCCCAGGTTGA

Primer Pair Name	Size	Location		Sequence
Tub2MN1007	61	Tub2 Promoter	-311.0	TTTTCTTGTTTTATTTATTTCAACCTGG<br>CCAGTTTTGAGGAGGATATCTTTA
Tub2MN1009	61	Tub2 Promoter	-291.0	CAACCTGGGCCTAACAGTAAA<br>AAATTCAGCGATTAAGTGCACCAG
Tub2MN1011	61	Tub2 Promoter	-271.0	AGATATCCTCCTCAAAACTGGTG<br>AAAAGAAGAGAAGCCAGAACAAATT
Tub2MN1013	61	Tub2 Promoter	-251.0	GTGCACTTAATCGCTGAATTTGT<br>GCCCATGGGGGGAATAAAGA
Tub2MN1017	61	Tub2 Promoter	-211.0	TTCTTTATTCCCCCCATGGGC<br>GTGACCCGGCGCCAAATTC
Tub2MN1019	61	Tub2 Promoter	-191.0	CCAAAAAAAAATAGTACTATCAGGAATT<br>GGTCACTGTACACGTATATCGT
Tub2MN1022	61	Tub2 Promoter	-171.0	AGGAATTTGGCGCCGGGTC<br>TTTCCTTGTGGCGTCGCCTA
Tub2MN1024	61	Tub2 Promoter	-151.0	CGATATACGTGTACAGTGACCT<br>TTTTTTCTGTTTTTTTCCTTTTTCCTTG
Tub2MN1028	61	Tub2 Promoter	-111.0	AAAGGAAAAAAACAGAAAAAACAACAAAA<br>AGATCTATTAAAGTTTTCGTGTTTGTT
Tub2MN1030	61	Tub2 Promoter	-91.0	ACAACAAAAACTAAAACAAACACGAAA<br>GCCTCACCACTACTTCACTTA
Tub2MN1032	61	Tub2 Promoter	-71.0	CACGAAAACTTTAATAGATCTAAGTGA<br>GCTGCTATGTCACTCCAATTG
Tub2MN1034	61	Tub2 Promoter	-51.0	TAAGTGAAGTAGTGGTGAGGCA<br>TTGCTTTTGTAGTTGTAGTAGCTG
Tub2MN1036	61	Tub2 Promoter	-31.0	CAATTGGAGTGACATAGCAGCT<br>TATATTACTTTGTGGAGATTTTGCTTT
Tub2MN1038	61	Tub2 Promoter	-11.0	CTACTACAACTACAAAAGCAAAATC<br>ATATGAATGATTTCTCTCATTATATTACT
Tub2MN1040	61	Tub2 ORF	10.0	AAATCTCCACAAAGTAATATAATGAGAG<br>TACCACACTGACCTGTCGAG
Tub2MN1042	61	Tub2 ORF	30.0	AATGAGAGAAATCATTCATATCTCG<br>GAATGCAGCACCAATTTGGTTAC
Tub2MN1046	61	Tub2 ORF	70.0	AACCAAATTGGTGCTGCATTCTG<br>CATTGAAATCCAAACCGTGCTC
Tub2MN1048	61	Tub2 ORF	90.0	CTGGGAAACTATCTGTGGTGA<br>GTCATGGCCGTGATATGTCC
Tub2MN1050	61	Tub2 ORF	110.0	AGCACGGTTTGGATTTCAATGG<br>AGTCTCTCCTTCTGGATATCGT
Tub2MN1053	61	Tub2 ORF	130.0	GGGACATATCACGGCCATG<br>CCTCGTTGAAGTACACGTTCA
Tub2MN1055	61	Tub2 ORF	150.0	CGATATCCAGAAGGAGAGACT<br>AACCCACTTCCCAGAAGATGC
Tub2MN1057	61	Tub2 ORF	170.0	TGAACGTGTACTTCAACGAGG<br>TCGACGTTAATAGATCTTGGAAC
Tub2MN1059	61	Tub2 ORF	190.0	GCATCTTCTGGGAAGTGGGT<br>CAATCGTCCCAGGTTCTAGAT

Primer Pair Name	Size	Location	Sequence
Tub2MN1061	61	Tub2 ORF 210.0	TCCAAGATCTATTAACGTCGATCTA<br>GGCAGAATTGCGTACTGCGT
Tub2orf02	61	Tub2 ORF 230.0	ATCTAGAACCTGGGACGATTG<br>GGTCTAAACAAATTCCCGATGG
Tub2MN1062	61	Tub2 ORF 830.0	TGGTCGGCTACGCTCCATT<br>GGGACAGTCAAAGATCTAAATGA
Tub2MN1064	61	Tub2 ORF 850.0	ACGGCAATTGGCTCTCAATCATTT<br>ACATTTGCTGTGTTAATTCAGGG
Tub2MN1066	61	Tub2 ORF 870.0	ATTTAGATCTTTGACTGTCCCTGAA<br>CATCATGTTCTTGGCATCAAACA
Tub2MN1068	61	Tub2 ORF 890.0	CTGAATTAACACAGCAAATGTTTGA<br>TTTCTTGGATCGGCAGCAGC
Tub2MN1073	61	Tub2 ORF 930.0	GGCTGCTGCCGATCCAAG<br>TTTACCTCTAAAGAAGGCTGCAA
Tub2MN1075	61	Tub2 ORF 950.0	ACGGTAGATACCTTACCGTTG<br>TCCACCTCCTTAACGGAAACTT
Tub2MN1077	61	Tub2 ORF 970.0	GCAGCCTTCTTTAGAGGTAAAG<br>GCACTTTATGCATTTCATCTTCC
Tub2MN1079	61	Tub2 ORF 990.0	AGTTTCCGTTAAGGAGGTGGA<br>ATAGTCTGAGTTTTTAGATTGCACT
Tub2MN1081	61	Tub2 ORF 1010.0	AAGATGAAATGCATAAAGTGCAATC<br>TTGGGGATCCATTCCACGAAAT
Tub2MN1083	61	Tub2 ORF 1030.0	CAATCTAAAAACTCAGACTATTTCG<br>ACACAGCAGTTTGCACATTGTTG
Tub2MN1085	61	Tub2 ORF 1050.0	TTTCGTGGAATGGATCCCCAA<br>ACCTTGAGGAGCGACAGAAC
Tub2MN1087	61	Tub2 ORF 1070.0	ACAATGTGCAAACTGCTGTGTG<br>AAAGTAGCAGCCATGTCCAAAC
Tub2MN1089	61	Tub2 ORF 1090.0	TGTTCTGTCGCTCCTCAAGG<br>TAGATGTGGAGTTAGCAATGAAAG
Tub2MN1091	61	Tub2 ORF 1110.0	TTTGGACATGGCTGCTACTTTC<br>TCTCTTGAATAGCTCTTGAATAGAT
Tub2MN1093	61	Tub2 ORF 1130.0	TCATTGCTAACTCCACATCTATTC<br>GCGGAAAATTGGTCACCAACT
Tub2MN1095	61	Tub2 ORF 1150.0	ATTCAAGAGCTATTCAAGAGAGTTG<br>AAGCTTTTCTTTTGAACATAGCGG
Tub2MN1097	61	Tub2 ORF 1170.0	AGTTGGTGACCAATTTTCCGC<br>ACTAGTATACCAGTGCAAGAAAGC
Tub2MN1099	61	Tub2 ORF 1190.0	CTATGTTCAAAAGAAAAGCTTTCTTG<br>TCCAATTCGTCCATACCTTCAC
Tub2MN1101	61	Tub2 ORF 1210.0	TTCTTGCACTGGTATACTAGTGAA<br>TAGATTCAGCCTCAGAGAATTCC
Tub2orf02	81	Tup1 ORF 2040.0	TGTTGCAAGGTCATAGGAATTCA<br>CAAAAACGTTATATTCTGGACCC
Gal2MN1041	61	Gal2p1 Promoter -571.0	GGCAACTACTTTGCATCAAACTC<br>GAGTACCTTTTGTGAAAAGATTCTA

Primer Pair Name	Size	Location	Sequence
Gal2MN1054	41	Gal2p1 Promoter -441.0	TCACGTGATCTATATTCGAAAGG<br>CTTCCTGAGGCAACCGCC
Gal2MN1085	61	Gal2p1 Promoter -191.0	TACAACATTCTGGAGAGCTATTG<br>TCTCCACATTTTAGCCTGCGA
Gal2MN1095	61	Gal2p1 Promoter -91.0	TGGATTGAAAATTTGGTGTTGTGAA<br>TGATGATAATTGAATAAGGTGCATAAT
Gal2MN1113	61	Gal2 ORF 1670.0	TTCCTTCATCCAGAAGAGGTAAT<br>GGTTTGTCGTCATGTTGTAAATC
Gal2MN1114	61	Gal2 ORF 1690.0	AATAATTACGATTTAGAGGATTTACAAC<br>CTAGCATGGCCTTGTACCAC
Rpb11orf03	61	Rpb11 ORF 320.0	AGCTTGGCGCCCTGAAGAC<br>TCGGCGGCTAGAGTTTGCA
